# Association of PFAS and Metals with Cardiovascular Disease Risk: Exploring the Mediating Effect of Diet

**DOI:** 10.3390/environments12060178

**Published:** 2025-05-28

**Authors:** Augustina Odediran, Kenneth Bollen, Emmanuel Obeng-Gyasi

**Affiliations:** 1 Department of Built Environment, North Carolina A&T State University, Greensboro, NC 27411, USA; 2 Environmental Health and Disease Laboratory, North Carolina A&T State University, Greensboro, NC 27411, USA; 3 Department of Psychology and Neuroscience, University of North Carolina at Chapel Hill, Chapel Hill, NC 27599, USA; 4 Department of Sociology, University of North Carolina at Chapel Hill, Chapel Hill, NC 27599, USA

**Keywords:** PFAS, metals, mixtures

## Abstract

**Background:**

Cardiovascular disease (CVD) is a major global health burden influenced by genetic, behavioral, and environmental factors. Among these, exposure to per- and poly-fluoroalkyl substances (PFASs) and toxic metals has been increasingly implicated in adverse cardiovascular outcomes. However, the mediating role of dietary inflammation in these associations remains unclear.

**Objective:**

This study investigates the relationship between PFAS and metal exposures and CVD risk, focusing on the potential mediating role of diet, operationalized through the Dietary Inflammatory Index (DII). Additionally, this study examines age as an effect modifier in these associations.

**Methods:**

Utilizing data from the National Health and Nutrition Examination Survey (NHANES) 2017–2018 cycle (*n* = 660), we assessed environmental exposures (lead, cadmium, mercury, perfluorooctanoic acid-PFOA, perfluorooctane sulfonate-PFOS), dietary inflammatory potential (DII), and cardiovascular markers (blood pressure, lipid profile, C-reactive protein). Statistical analyses included linear regression and Bayesian Kernel Machine Regression-Causal Mediation Analysis (BKMR-CMA) to estimate the direct, indirect (through DII), and total effects of exposure on CVD risk biomarkers.

**Results:**

Linear regression revealed significant associations between mercury and reduced systolic blood pressure (SBP) (*p* = 0.017) and cadmium with increased C-reactive protein (CRP) (*p* = 0.006). Mediation analysis suggested dietary inflammation may play a role, though estimates were imprecise.

**Conclusions:**

PFAS and metals may influence CVD risk through inflammatory pathways, with potential age-related differences. Future longitudinal studies are needed to clarify these complex interactions, reduce measurement error, and guide age-specific exposure regulations.

## 1. Introduction

Cardiovascular disease (CVD) encompasses a range of disorders affecting the heart and blood vessels, including coronary artery disease (CAD), hypertension, heart failure, and stroke. The underlying pathophysiological mechanisms often involve atherosclerosis, characterized by the build-up of fatty deposits, cholesterol, and other substances in the arterialwalls, leading to narrowing and reducing blood flow and oxygen delivery to vital organs [1]. Chronic inflammation is pivotal in the progression of atherosclerosis and is triggered by oxidative stress, endothelial dysfunction, and immune cell activation [2]. Hypertension, another critical component of CVD, results from increased vascular resistance and can exacerbate atherosclerosis and heart failure by imposing additional strain on the heart and blood vessels [3].

CVD is a leading cause of morbidity and mortality worldwide, accounting for approximately 17.9 million deaths annually, representing 32% of all global deaths, and a significant health and economic burden [4]. The staggering statistics highlight the significant health and economic burden posed by CVD. The development of CVD is complex and driven by the interplay of genetic, behavioral, and environmental factors that influence an individual’s risk. Among these, environmental exposures, particularly per- and polyfluoroalkyl substances (PFASs) and toxic metals, have garnered increasing attention due to their prevalence in industrialized environments and potential adverse health effects [5]. PFASs, commonly used in non-stick cookware, water-repellent clothing, food packaging, such as grease-resistant paper and cardboard, and firefighting foams, are highly persistent in the environment and the human body. Known for their long biological half-lives, PFASs have been associated with various health outcomes, including endocrine disruption, immune system impairment, and metabolic disturbances, each of which may contribute to elevated CVD risk [6]. Similarly, exposure to toxic metals, such as lead, cadmium, and mercury, primarily released through industrial activities, mining, and agricultural practices, has been shown to increase oxidative stress, disrupt lipid metabolism, and induce endothelial dysfunction—key pathways implicated in the pathogenesis of CVD [7].

Exposure to PFAS and heavy metals has been associated with adverse cardiovascular outcomes, including hypertension, atherosclerosis, and increased risk of CVD mortality [8,9]. Mechanistic studies suggest that these pollutants disrupt endocrine function, promote oxidative stress, and trigger systemic inflammation, all of which are critical pathways in the pathogenesis of CVD [10–12]. PFAS, for instance, can interfere with lipid metabolism, leading to dyslipidemia, which is a well-established risk factor for cardiovascular disease [13]. Similarly, heavy metals, such as lead and cadmium, are known to damage vascular endothelial cells and alter arterial compliance [14,15].

Despite the growing body of evidence linking these exposures to cardiovascular outcomes, the precise biological pathways and the role of modifiable factors remain inadequately understood. Studies exploring the cumulative effects of PFAS and metals on cardiovascular health remain limited, particularly those that account for the interaction between these exposures and modifiable lifestyle factors [16]. Given the growing evidence of cardiovascular toxicity associated with both PFAS and heavy metals, our study focuses on five widely studied and environmentally persistent pollutants: perfluorooctanoic acid (PFOA), perfluorooctane-sulfonic acid (PFOS), lead (Pb), cadmium (Cd), and mercury (Hg) [17,18]. These compounds were selected based on their high detection frequency in the U.S. population (as observed in NHANES), established toxicological profiles, and consistent associations with cardiovascular outcomes in epidemiologic and mechanistic studies. Importantly, these pollutants are prioritized by regulatory agencies such as the U.S. Environmental Protection Agency (EPA) and the Agency for Toxic Substances and Disease Registry (ATSDR) due to their widespread human exposure and health relevance. By analyzing both PFAS and metals together, this study captures a mixture of organic and inorganic contaminants commonly encountered in industrialized environments [19,20]. Traditional environmental health research has often focused on assessing the effects of individual pollutants in isolation. While this approach has yielded important insights, it does not reflect the reality of human exposure, where individuals are simultaneously subjected to complex mixtures of chemicals [21]. Co-exposure to PFAS and metals may result in additive, synergistic, or antagonistic effects that are not detectable through single-pollutant models. To more accurately characterize these combined risks, it is essential to evaluate exposures jointly using mixture modeling frameworks. This enables the identification of non-linear interactions, estimation of joint dose–response relationships, and differentiation between cumulative and independent effects. By studying pollutants like PFOA, PFOS, Pb, Cd, and Hg in a single model, we enhance our ability to interpret real-world exposure profiles and inform more relevant regulatory and public health strategies. Our selection strategy also considered data availability and statistical modeling limitations to ensure robust estimation without overfitting, particularly in the context of Bayesian Kernel Machine Regression with Causal Mediation Analysis (BKMR-CMA). Integrating this pollutant mixture framework with a focus on dietary inflammation allows us to investigate modifiable pathways that may mitigate the cardiovascular impacts of environmental exposures. Owing to the challenges in controlling environmental exposure, understanding how diet—an accessible and modifiable factor—may interact with or even mediate the effects of these toxicants on cardiovascular health could be critical.

Dietary patterns, especially those high in antioxidants, fiber, and anti-inflammatory nutrients, have been shown to mitigate the harmful effects of oxidative stress and systemic inflammation, which are common pathways through which PFAS and metals exert their toxic effects [22,23]. Conversely, diets rich in saturated fats, refined sugars, and processed foods are known to exacerbate inflammation and oxidative stress, potentially heightening an individual’s susceptibility to the adverse cardiovascular impacts of environmental toxicants [24]. Moreover, certain dietary components, such as calcium, iron, and selenium, may influence the body’s ability to absorb, metabolize, and eliminate toxic metals, thereby potentially modifying the risk associated with exposure [25]. The role of diet as a potential mediator in the relationship between PFAS, metals, and cardiovascular health remains underexplored, representing a significant gap in understanding how environmental and lifestyle factors intersect to influence CVD risk.

The Dietary Inflammatory Index (DII) is a quantitative measure that assesses the inflammatory potential of an individual’s diet [26]. The DII is calculated based on the intake of various dietary components, including macronutrients, micronutrients, and specific food items, which are then scored against a standardized global database. Foods with high DII scores, such as processed meats, refined carbohydrates, and sugary beverages, are associated with a pro-inflammatory response. Conversely, foods with low DII scores, such as fruits, vegetables, nuts, and whole grains, are linked to anti-inflammatory effects. This index enables researchers to quantify the relationship between dietary patterns and inflammation in epidemiological studies. Emerging evidence suggests that the DII may be a critical mediator in the relationship between environmental exposures and CVD risk [27]. A diet high in inflammatory potential may exacerbate the effects of PFAS and heavy metals by amplifying systemic inflammation and oxidative stress. Conversely, an anti-inflammatory diet may mitigate these effects by enhancing detoxification pathways and reducing pollutant-induced oxidative damage [28]. Supporting this, Labaronne and colleagues [29] found that low-dose pollutant exposure and a high-fat diet independently disrupted similar hepatic metabolic pathways in mice—including those related to lipid metabolism and inflammation—underscoring the diet’s role in modulating pollutant-related health effects.

This study aims to investigate the association between PFAS and metal exposures and CVD risk, focusing on evaluating the mediating effect of diet in this relationship. By examining the joint effects of environmental toxicants and dietary patterns, we seek to contribute to a more nuanced understanding of the determinants of cardiovascular health. Specifically, this study will explore whether individuals with healthier nutritional patterns experience reduced cardiovascular risks associated with PFAS and metal exposures compared to those with less healthy diets. The findings from this research may inform the development of targeted dietary recommendations and public health interventions that address environmental exposures and lifestyle factors as part of comprehensive strategies to reduce cardiovascular risk.

In addition to advancing scientific knowledge, this research may have practical implications for mitigating the impacts of environmental pollution on cardiovascular health. Insights into the dietary modulation of PFAS and metal toxicity could guide policies aimed at reducing exposure in vulnerable populations, such as those living near industrial areas or consuming elevated levels of contaminated food and water. Ultimately, this study underscores the importance of a holistic approach to understanding CVD risk, recognizing the interconnected roles of environmental, biological, and lifestyle factors in shaping individual health outcomes.

## 2. Materials and Methods

### 2.1. Study Population and Design

Data for this study were derived from the National Health and Nutrition Examination Survey (NHANES), a nationwide survey conducted by the U.S. Centers for Disease Control and Prevention (CDC) in two-year cycles, representing the non-institutionalized U.S. population. Using a complex, multi-stage sampling approach, NHANES provides valuable data on health and nutritional indicators across the United States. For this study, we utilized data from the NHANES 2017–2018 cycle, encompassing participants from all 50 states and the District of Columbia. Survey participants underwent physical examinations and interviews, with blood samples collected for laboratory analysis. The Centers for Disease Control and Prevention measured PFAS and PFOS in the serum of NHANES participants aged 12 and above. The metal (cadmium, mercury, and lead) content in whole blood specimens was directly measured using Inductively Coupled Mass Spectrometry (ICP-MS) following a simple dilution sample preparation step (ICP-MS; CDC method No. ITB0001A). The blood metals were analyzed using a PerkinElmer NexION 300D ICP-MS (PerkinElmer, Inc., Waltham, MA, USA). Detailed documentation on NHANES survey methods and procedures is available on the CDC’s website [30].

### 2.2. Calculation of Dietary Inflammatory Index (DII) Scores

To assess dietary inflammation, we calculated individual Dietary Inflammatory Index (DII) scores based on 24 h dietary recall interviews using Hébert’s method [26,31]. The DII assesses the inflammatory potential of diet by evaluating 45 dietary components; in this study, NHANES 2017–2018 data included 27 components. Key nutrients used in DII calculation included carbohydrates, proteins, fats, cholesterol, vitamins, minerals, and other dietary elements, such as thiamine, riboflavin, beta-carotene, and folic acid. Each participant’s DII was averaged across dietary recalls to create a stable measure of dietary inflammation. Previous studies have validated DII calculations even when fewer dietary components are available.

### 2.3. Statistical Analysis

This analysis utilized complete data only, excluding cases with missing values, to maintain data integrity without the need for imputation. We conducted initial data cleaning to ensure consistency and accuracy within the dataset.

The primary analytical methods included linear regression and Bayesian kernel machine regression (BKMR). Linear regression explored direct associations between predictor variables and cardiovascular outcomes, while BKMR captured potential non-linear relationships and interactions between pollutants. The combination of these methods provided a comprehensive perspective on the data.

#### 2.3.1. Descriptive Statistics

We calculated descriptive statistics to summarize the distributions of PFAS, metals, and demographic variables, stratifying by DII scores. Spearman correlations were used to assess associations among variables due to their ability to capture monotonic relationships.

#### 2.3.2. Bayesian Kernel Machine Regression

BKMR was applied to evaluate the combined impact of PFAS and metals on cardiovascular risk, specifically focusing on perfluorooctanoic acid (PFOA), perfluorooctane sulfonate (PFOS), mercury, lead, and cadmium. Following the methodology of Bobb et al. [21], we used Markov chain Monte Carlo (MCMC) sampling for 5000 iterations. Posterior inclusion probabilities (PIPs) helped assess the influence of each contaminant in the mixture.

To visualize exposure–response relationships, we calculated high-dimensional functions for various exposure levels, holding other covariates constant at median values. This enabled a comparative analysis of pollutant effects on cardiovascular outcomes across exposure percentiles.

The model was structured as follows:

$$\text{g}\left({\text{μ}}_{\text{i}}\right)\;\text{= h}\left({\text{z}}_{\text{i}}\text{1, …,}{\text{z}}_{\text{i}}\text{M}\right)\;\text{ + β}{\text{X}}_{\text{i}}\text{;i = 1, …,n}$$

where g is a link function, ${\text{μ}}_{\text{i}}=\text{E}\left({\text{Y}}_{\text{i}}\right)$ is the health end point; h represents the flexible kernel function of exposures ${\text{z}}_{\text{i}}\text{1,…,}{\text{z}}_{\text{i}}\text{M}$; x is the vector of covariates for the i-th observation, covariates such as BMI, gender, age, ethnicity, alcohol use and smoking status

#### 2.3.3. Mediation Analysis

Causal questions seek to assess whether the world would change if observations were exposed to one state of the world compared to another. The key idea is that there is a causal variable—sometimes called a treatment—which must be something that can be changed in the real world. These questions allow for understanding how interventions impact health outcomes. Essentially, we compare two versions of the world: one where the exposure occurs and another where it does not. The difference between these two worlds defines the counterfactual—a hypothetical scenario that allows us to estimate causal effects.

In the normal setting, we observe individuals in their actual environment:

- Their exposure (A) to pollutants like PFAS and metals.
- Their mediator (M), in this case, diet, could influence the relationship between exposure and health.
- Their outcome (Y), such as cardiovascular risk, is what we are ultimately interested in understanding.

This is the standard epidemiological framework where we analyze how exposure and mediators relate to the outcome.

In the counterfactual setting, we consider what would have happened to the outcome if we could manipulate the exposure (A) and mediator (M) to different levels. This hypothetical comparison allows us to answer causal questions:

- What would an individual’s life look like if they were exposed to lower levels of PFAS and metals?
- Would their cardiovascular risk be lower?
- How much of this effect would be due to dietary changes?

The counterfactual world helps us define and estimate causal effects by considering these hypothetical scenarios.

To formally describe these causal effects, we use counterfactual notation:

- ${\text{Y}}_{\text{a}}$: The counterfactual outcome Y if exposure A were set to level a.
- ${\text{M}}_{\text{a}}$: The counterfactual mediator M that would have been observed if exposure A were set to a.
- ${\text{Y}}_{\text{a}}{\text{M}}_{{\text{a}}^{\text{*}}}$: The counterfactual outcome when exposure is set to **a** and the mediator is set to the level it would have taken under exposure ${\text{a}}^{*}$.

To explore the role of diet in mediating the relationship between PFAS and metals with cardiovascular risk, we employed Bayesian Kernel Machine Regression-Causal Mediation Analysis (BKMR-CMA). This approach allows for the estimation of complex, potentially nonlinear, and interactive relationships between the exposure mixture, mediator, and outcome. Using the counterfactual framework, we decompose the total effect (TE) into the natural direct effect (NDE) and the natural indirect effect (NIE) to distinguish how much of the effect of exposure on the outcome is mediated through diet:

$$\text{TE = E}\left[{\text{Y}}_{\text{a}}\;\text{-}\;{\text{Y}}_{{\text{a}}^{\text{*}}}\right]$$

This represents the overall impact of changing exposure from ${\text{A}}^{*}$ to A on the outcome Y.


$$\text{NDE = E}\left[{\text{Y}}_{\text{a}}{\text{M}}_{{\text{a}}^{\text{*}}}\;\text{-}\;{\text{Y}}_{{\text{a}}^{\text{*}}}{\text{M}}_{{\text{a}}^{\text{*}}}\right]$$

This captures the effect of changing exposure from ${\text{A}}^{*}$ to A, while holding the mediator constant at the level it would have taken under ${\text{A}}^{*}$. In other words, this represents the direct effect of exposure on health, independent of diet.


$$\text{NIE = E}\left[{\text{Y}}_{\text{a}}{\text{M}}_{\text{a}}\;\text{-}\;{\text{Y}}_{\text{a}}{\text{M}}_{{\text{a}}^{\text{*}}}\right]$$

This measures the portion of the total effect that operates through the mediator (diet). It captures how much of the effect of exposure on the outcome is due to changes in diet rather than direct effects of the pollutant.

To model these effects, we specified separate BKMR models for the mediator and outcome:

$$M_{i}=h_{M}\left(Z_{Mi}\right)+C_{i}^{T}\beta +\varepsilon_{Mi}$$

$$Y_{i}=h_{Y}\left(Z_{Yi},M_{i}\right)+C_{i}^{T}\text{θ}+\varepsilon_{Yi}$$

where $h_{M}(\cdot )$ and $h_{Y}(\cdot )$ are flexible functions that account for nonlinear associations and interactions among the exposure mixture and mediator, and $C_{i}^{T}$ represents additional covariates. We further assessed the controlled direct effect (CDE), which quantifies the exposure-outcome relationship when the mediator is fixed at specific values:

$$\text{CDE(m) = E}\left[{\text{Y}}_{\text{am}}\;\text{-}\;{\text{Y}}_{{\text{a}}^{\text{*}}\text{m}}\right]$$

Age was tested as an effect modifier in these relationships to determine whether the mediation effects varied across different age groups.

The analysis was conducted in R (version 4.2.3) with a significance level of 0.05 for non-Bayesian analyses.

## 3. Results

### 3.1. Descriptive Statistics of Study Sample

A total of 660 participants were included in this study. The mean age of the participants was 49.33, and the standard deviation (SD) was 18.51. The average body mass index (BMI) was 29.80 kg/m^2^, with a standard deviation of 7.79. Other characteristics of the population, such as environmental exposure biomarkers (lead, cadmium, PFOA, PFOS, mercury), dietary inflammatory index, and cardiovascular risk factors (for example, SBP, DBP, total cholesterol, triglycerides, C-reactive proteins, LDL cholesterol, HDL cholesterol), are summarized in Table 1.

### 3.2. Linear Regression of Cardiovascular-Related Markers and Key Study Variables

Table 2 summarizes the results of the linear regression, which examined the association between SBP and various PFAS and metals, and the Dietary Inflammatory Index. Mercury demonstrated a statistically significant negative association with SBP, indicating that higher levels of mercury are associated with reduced systolic Blood Pressure (SBP) (β = −0.948, *p* = 0.017, 95% CI: −1.723, −0.172). PFOA exhibited a marginally significant positive association (β = 1.025, *p* = 0.095, 95% CI: −0.178, 0.0934). Other pollutants, including lead, cadmium, PFOS, and DII, did not exhibit a statistically significant association with SBP.

### 3.3. Spearman Correlation Analysis of Key Study Variables

**Figure 1.**
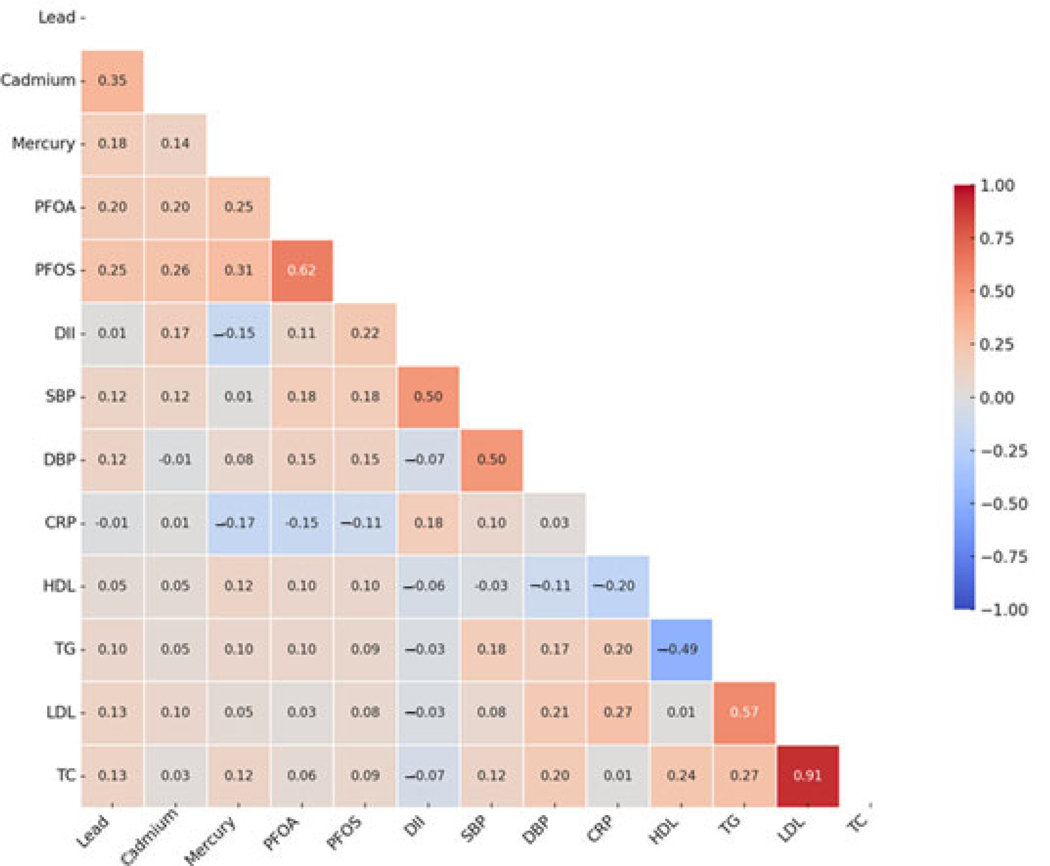
Spearman correlation plot of critical study variables.

Figure 1 illustrates the Spearman correlation analyses conducted among the exposure variables, Dietary Inflammatory Index (DII), and outcome variables. Strong correlations are represented by larger circles and more vibrant colors, with blue indicating positive associations and red indicating negative associations.

### 3.4. Bayesian Kernel Machine Regression Causal Mediation Analysis

BKMR-CMA was used to estimate the risk Summary for the Total Effect, examining how the combined impact of all exposures on cardiovascular-related outcome variable levels varied across different age groups, highlighting potential changes in risk with age.

#### Systolic Blood Pressure

**Figure 2.**
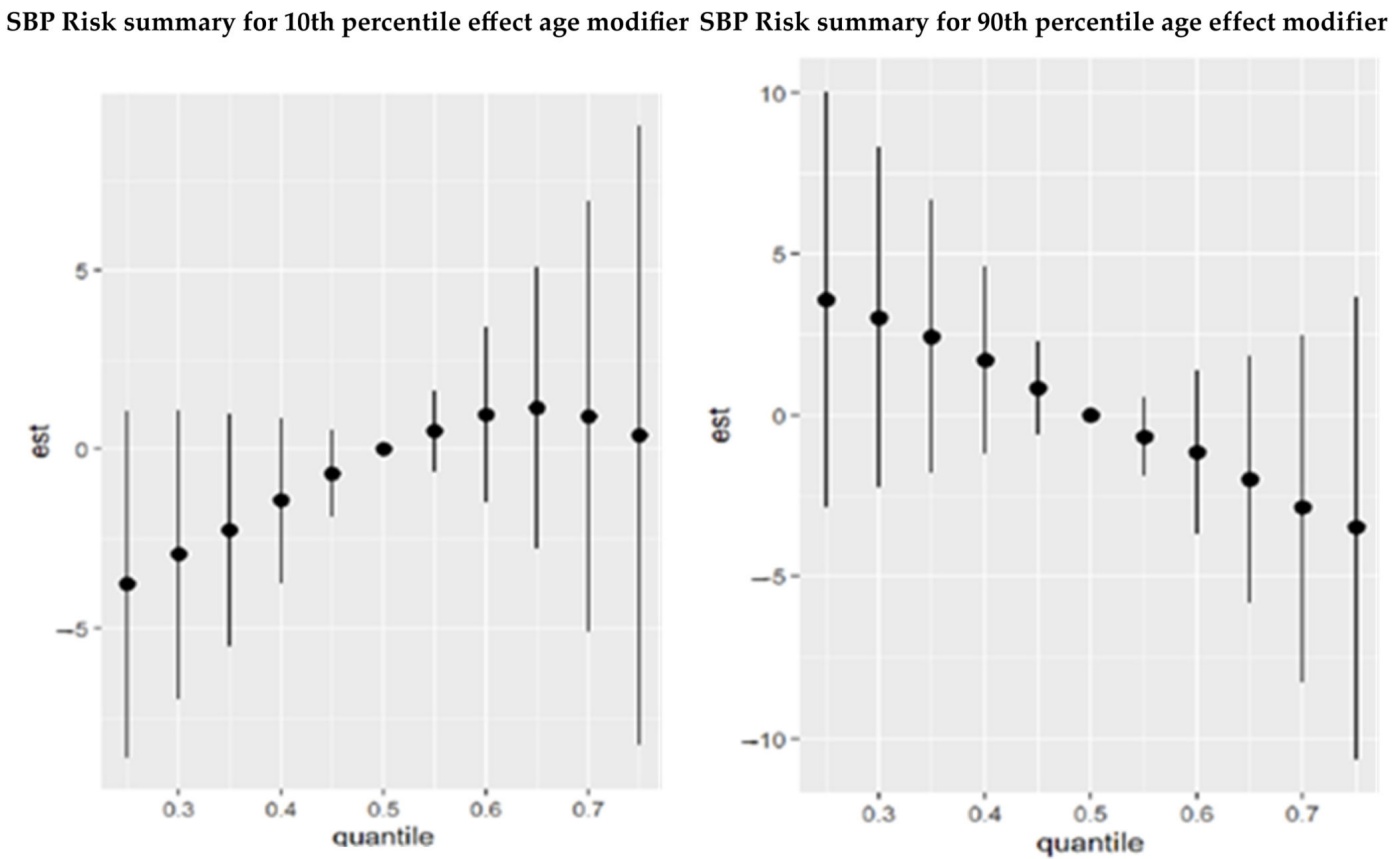
The overall exposure effect of combined PFAS and metals on SBP was examined at 0.25–0.75 quantiles of exposure as compared to the 0.5 quantile.

The combined effects of PFAS and metals on SBP, as analyzed using BKMR, revealed the following (Figure 2):

- Younger Age Group (10th Percentile): For younger individuals, the combined exposure to PFAS and metals has varying effects that become more positive at higher exposure levels for younger individuals. The plot shows an increasing trend from negative to slightly positive values across quantiles, but large credible intervals that cross zero emphasize the uncertainty in the results.
- Older Age Group (90th Percentile): For older individuals, the combined exposure to PFAS and metals shows a consistent negative association with SBP at higher exposure levels for older individuals suggesting the negative effects are at lower quantiles of exposure. The wide credible intervals that cross zero indicate an elevated level of uncertainty in this group.

This age-dependent pattern highlights that PFAS and metals might influence SBP differently depending on age, with older individuals potentially more sensitive to exposure effects on blood pressure.

**Figure 3.**
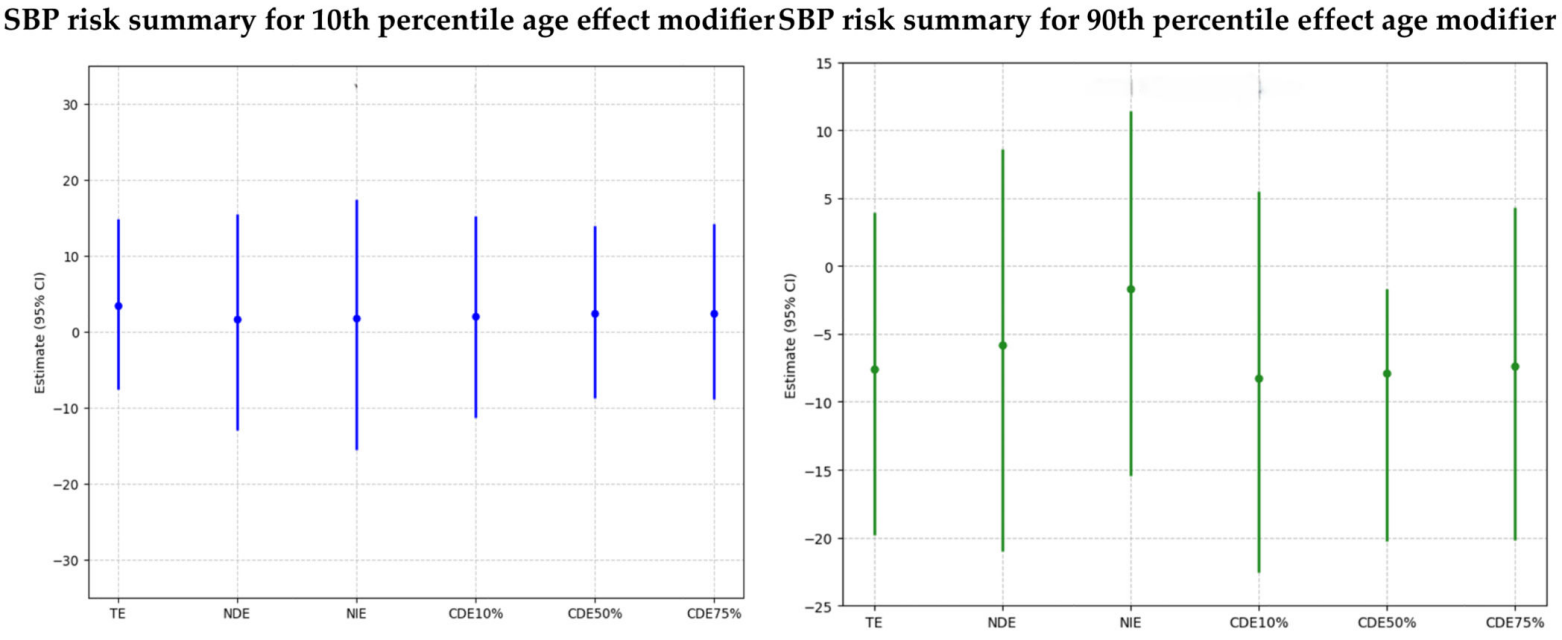
Causal mediation analysis assessing the combined effect of PFAS and metals on SBP with DII as mediator.

BKMR-CMA was also used to estimate the direct and indirect effects of PFAS and metal mixtures on SBP through the DII level according to age (Figure 3). The results were as follows:

Left Plot: 10th Percentile of Age (Younger Age Group)

- TE (Total Effect): The total effect, combining both direct and mediated pathways, shows a minor positive effect in SBP associated with exposure in younger individuals, but this effect is not strong, with the large credible interval crossing zero, emphasizing the uncertainty in the results.
- NDE (Natural Direct Effect): This reflects the direct impact of PFAS and metals on SBP, independent of the DII. The slightly positive estimate suggests a direct association between PFAS/metals and SBP, though the effect is weak and uncertain due to an overlapping credible interval that crosses zero.
- NIE (Natural Indirect Effect): This shows the impact of PFAS and metals on SBP that is mediated through the DII. The estimate is close to zero, suggesting that, for younger individuals, the inflammatory diet does not mediate the relationship between PFAS/metals and SBP.
- CDEs at Different Quantiles of DII (10%, 50%, 75%): The Controlled Direct Effects remain close to zero or slightly positive, indicating that, even when controlling for specific levels of DII, PFAS, and metals, they do not have a strong impact on SBP in younger individuals. Additionally, the large, credible intervals, which also cross zero, highlight the uncertainty in the results.

Right Plot: 90th Percentile of Age (Older Age Group)

- TE (Total Effect): The total effect of PFAS and metals on SBP is negative, suggesting a potential reduction in SBP with exposure. Nevertheless, the credible interval crosses zero, highlighting the uncertainty in the results.
- NDE (**Natural Direct Effect**): The negative direct effect estimate suggests a higher PFAS/metals lower SBP. The slightly larger magnitude of NDE indicates that the observed effect might be due to the direct pathway. The credible interval is large and crosses zero, highlighting the results’ uncertainty.
- NIE (Natural Indirect Effect): The indirect effect is negative, showing that DII negatively and minimally mediates the impact of PFAS and metals on SBP for older individuals. Additionally, the credible interval is large and crosses zero, highlighting the results’ uncertainty.
- CDEs at Different Quantiles of DII (10%, 50%, 75%): The Controlled Direct Effects show a negative trend, with effects being slightly negative, indicating that exposure effects on SBP are negative in older individuals across PFAS/metal exposure levels. Additionally, the credible intervals are large and cross zero, highlighting the uncertainty in the results.

The results suggest that the Dietary Inflammatory Index (DII) does not strongly mediate the relationship between PFAS/metal exposure and SBP at the extremes of age, as assessed at the 10th and 90th percentiles of age. While the effects show some variation, with younger individuals (10th percentile) potentially experiencing a slight increase in SBP and older individuals (90th percentile) a minor decrease, the wide credible intervals crossing zero indicate considerable uncertainty in these estimates. This implies that DII is not a meaningful mediator of the relationship between PFAS/metals and SBP, and age, as an effect modifier, does not appear to substantially alter this mediation pathway in this analysis.

Additional linear regression and BKMR-CMA results examining the associations between PFAS, metals, DII, and cardiovascular biomarkers—including DBP, HDL, LDL, total cholesterol, CRP, and triglycerides—are presented in the Supplementary Material. These analyses explore age-specific trends and mediation effects, with most associations showing high uncertainty.

## 4. Discussion

This study investigated the association between individual and combined exposure to PFAS and metals and CVD risk. We focused on evaluating the mediating effects of diet operationalized through the Dietary Inflammatory Index. Using data from the National Health and Nutrition Examination Survey, we applied linear regression and Bayesian Kernel Machine Regression-Causal Mediation Analysis to assess the direct and indirect effects of the combined exposure to PFAS and heavy metals on cardiovascular outcomes, applying age as an effect modifier to evaluate its potential differential role among those in the 10th and 90th percentile age groups [32]. Our findings add to the existing literature exploring the association between environmental exposure, particularly PFAS, and heavy and cardiovascular disease risk markers. Although most observed associations in our analyses were modest and did not reach statistical significance—evidenced by wide credible intervals consistently overlapping zero—the trends noted align with prior studies suggesting potential biological pathways connecting these pollutants with cardiovascular health outcomes. Understanding the interplay between environmental exposure, dietary inflammation, and cardiovascular disease is critical for public health and medical sciences.

The linear regression analysis revealed a complex association between combined exposure to PFAS, heavy metals, and various cardiovascular biomarkers. Among these associations, mercury exhibited a significant negative association with SBP, suggesting that higher mercury exposure was linked to reduced SBP, a finding that appears counterintuitive. This unexpected negative association could be influenced by dose-dependent effects, confounding factors, or physiological mechanisms. While mercury is typically associated with adverse cardiovascular effects, prior research has reported inconsistent findings, with some studies finding no definitive association between mercury and cardiovascular disease (CVD). In contrast, others suggest a positive association [33]. These discrepancies may arise due to differences in study populations, exposure levels, and confounding factors, underscoring the need for further research to clarify the relationship. One important potential confounder in this association is dietary intake of omega-3 fatty acids, particularly from fish consumption. Mercury exposure is commonly linked to the consumption of fish and seafood, which are also rich sources of eicosapentaenoic acid (EPA) and docosahexaenoic acid (DHA), two omega-3 fatty acids known for their cardioprotective effects, including reductions in blood pressure and inflammation [34]. It is possible that individuals with higher mercury exposure also have higher omega-3 intake, which may counteract some of the expected adverse cardiovascular effects of mercury and contribute to the observed negative association with SBP. This dietary factor introduces a potential confounding effect that should be carefully considered in future studies.

Conversely, PFOA showed a marginally significant positive association with SBP, consistent with previous studies. PFOSs are linked to adverse cardiovascular health in both young and middle-aged groups [28]. Various studies have suggested that PFAS may contribute to hypertension by promoting vascular inflammation, oxidative stress, and endothelial dysfunction [35,36].

Cadmium exposure was significantly associated with increased CRP levels, an established marker of systemic inflammation. Previous studies have shown that cadmium is linked to elevated CRP levels. An elevated cadmium level induces oxidative stress, activates pro-inflammatory signaling pathways, and induces cellular damage and inflammatory responses, critical pathways in CVD pathogenesis [37]. The relationship between cadmium and CRP may be dose-dependent [38,39]. There is a relationship between cadmium and CVD mortality [40]. Additionally, DII demonstrated a borderline significant positive association with CRP, reinforcing the notion that pro-inflammatory dietary patterns may exacerbate systemic inflammation, potentially amplifying the adverse cardiovascular effects of environmental toxicants [26,27]. Pro-inflammatory dietary patterns include Western-type diets that are high in red meat, high-fat dairy products, refined grains, and simple carbohydrates, and these are linked to higher levels of CRP [41].

The relationship between heavy metals, specifically mercury, and perfluoroalkyl substances (PFASs), such as perfluorooctanoic acid (PFOA), with lipid profiles and cardiovascular disease (CVD) risk is complex and warrants further investigation. Our study indicated that mercury exposure is significantly associated with higher levels of high-density lipoprotein (HDL) cholesterol, while PFOA negatively correlates with HDL levels. This paradoxical finding regarding mercury suggests a potential protective role in lipid metabolism despite its well-documented toxic effects [42,43]. Conversely, the inverse relationship between PFOA and HDL aligns with the existing literature that posits PFAS disrupts lipid homeostasis through alterations in peroxisome proliferator-activated receptor (PPAR) signaling pathways [44,45].

The observed association between mercury and elevated HDL levels is intriguing as it contrasts with the widely recognized harmful effects of mercury on cardiovascular health. While mercury exposure is associated with various adverse outcomes, its correlation with increased HDL may reflect a more complex interaction that warrants further investigation [42,43]. One plausible explanation is the influence of dietary patterns, particularly fish consumption, as fish are a common source of both mercury and beneficial omega-3 fatty acids. Omega-3s are well-documented for their role in raising HDL levels and supporting cardiovascular health, potentially offsetting some of the negative effects of mercury. Therefore, the observed positive association may not represent a direct beneficial effect of mercury itself, but rather a confounded relationship mediated by diet [46–49].

On the other hand, the negative association between PFOA and HDL levels is consistent with findings that PFAS can lead to dyslipidemia, characterized by increased total cholesterol and LDL levels, alongside decreased HDL [50,51]. The disruption of PPAR signaling by PFAS has been implicated in these lipid alterations, suggesting that exposure to these substances could lead to a pro-inflammatory state that exacerbates cardiovascular risks [44,45].

The lack of significant associations between PFAS/metals and LDL and total cholesterol suggests that the impact of these exposures on lipid metabolism may be complex and dependent on additional factors, such as genetic predisposition and dietary habits [52,53].

### 4.1. The Mediating Role of Diet

One of the critical contributions of this study is its exploration of the mediating effect of diet on the association between environmental pollutants, such as PFAS and heavy metals, and CVD risks, a significant advancement in understanding the complex interplay between dietary patterns and environmental exposure. The BKMR-CMA results provided nuanced insights into the mediating role of diet in the PFAS/metals–CVD relationship. Across multiple cardiovascular biomarkers, DII did not appear to significantly mediate the effects of PFAS and heavy metals on cardiovascular outcomes. Notably, the results suggest that, while diet influences inflammation and CVD risk, it may not substantially modify the toxicological effects of these environmental exposures.

A study conducted by Odediran and Obeng-Gyasi highlights the association between combined metals and PFAS exposure with dietary patterns, suggesting that a pro-inflammatory diet may act as a mediator between environmental exposure and chronic diseases, including CVD [54]. However, the BKMR-CMA results reveal that the DII played a differential role in mediating the effects of PFAS and heavy metals on cardiovascular outcomes, suggesting that diet plays a complex role in how pollutants affect different cardiovascular-related markers. Our findings suggest that DII may not play a major role in the relationship between PFAS/metals exposure and CVD. Similarly, the work of Trautwein and McKay highlights that specific dietary patterns—particularly those rich in whole grains and low in refined sugars—can help mitigate cardiovascular risk but may not fully counteract the adverse effects of environmental pollutants [55].

The potential mediating role of diet proved to be subtler than anticipated, revealing important patterns across different cardiovascular outcomes. Stronger potential mediating effects were observed in younger individuals for inflammatory markers, while lipid parameters exhibited variable mediation patterns. Nevertheless, there was significant uncertainty, as all credible intervals crossed zero, indicating that the mediating effects were not strongly supported by the data.

These findings suggest that dietary inflammation may influence cardiovascular risk factors differently across age groups, but its mediating role in the relationship between PFAS/metals exposure and cardiovascular outcomes remains uncertain. Further research is needed to clarify these interactions, particularly with larger sample sizes and refined exposure assessments [56,57]. The age-specific nature of these potential mediating effects suggests the possibility of targeted dietary interventions to mitigate the cardiovascular impacts of environmental exposures. However, given the uncertainty in the estimates, as indicated by credible intervals crossing zero, further research is needed to confirm these effects and determine the effectiveness of such interventions.

For instance, younger individuals may experience a more pronounced inflammatory response to environmental pollutants, which their dietary habits could potentially influence. However, given the uncertainty in our estimates, this relationship should be interpreted cautiously. This observation aligns with the existing literature, suggesting that younger populations may be more susceptible to the inflammatory effects of dietary patterns and environmental exposures, but further research is needed to confirm these findings and establish causal links [58]. The variability in lipid parameters, such as high-density lipoprotein (HDL) and triglycerides (TGs), further underscores the complexity of these interactions. Research has shown that dietary patterns can significantly influence lipid metabolism, and these effects may differ based on age and exposure levels [56].

These findings have important but uncertain implications for public health interventions and risk assessment strategies. Given that the Dietary Inflammatory Index (DII) did not show a strong mediating effect, as all credible intervals crossed zero, caution is needed in interpreting its role. The observed age-dependent patterns suggest, but do not confirm, the need for age-specific exposure guidelines and intervention approaches.

The complex interaction between dietary factors and environmental exposures hints at the potential benefit of combined approaches addressing both exposure reduction and nutritional status, rather than single-focus interventions. However, the lack of strong statistical support underscores the need for further research before implementing targeted dietary strategies. While promoting the consumption of anti-inflammatory foods and discouraging high DII dietary patterns could theoretically help mitigate cardiovascular risks associated with environmental pollutant exposure, their effectiveness remains uncertain. Early-life interventions may warrant further exploration, given the different patterns of response observed in younger individuals, but additional evidence is needed to support targeted recommendations.

### 4.2. Limitations

While BKMR-CMA accounts for nonlinear relationships, interactions, and mediation effects, several limitations remain. First, valid causal interpretation requires no unmeasured confounding between exposure, mediator, and outcome, an assumption that cannot be fully tested in NHANES. While gender was included as a covariate to control for confounding, we did not investigate it as an effect modifier due to sample size limitations and the complexity of the BKMR framework. Stratifying by both age and gender would have reduced the statistical power. Second, reliance on single-time-point biomarker measurements may not capture long-term exposure and dietary inflammation patterns, potentially biasing mediation estimates. Third, mediation effects depend on the choice of counterfactual exposure and effect modifier levels, requiring careful interpretation within the observed data range. While Bayesian methods provide credible intervals, estimates may be sensitive to prior specifications and MCMC convergence, warranting further sensitivity analyses. This study also did not account for the presence of infectious diseases, which may have affected CRP levels. Acute infections can raise CRP levels, potentially confounding interpretations of it as a marker of chronic inflammation. Finally, an additional limitation not fully addressed in the analyses is the assumption of the absence of measurement error in exposure assessment. Measurement error can lead to biased effect estimates and reduced statistical power, complicating the estimation of causal relationships. Future studies incorporating longitudinal data or interventions would strengthen causal inference. Accounting for measurement errors in dietary assessment, exposure estimates, and outcome variables will be crucial to improving the reliability and validity of findings. Future studies with larger sample sizes are warranted to investigate potential sex-based differences in susceptibility to combined PFAS and metal exposures in relation to cardiovascular outcomes. Furthermore, since CRP can be elevated due to acute infections, future analyses should adjust for systemic inflammation to better isolate the impacts of environmental exposures on inflammation.

### 4.3. Future Directions

Looking forward, several critical areas deserve further investigation. Given the observed trends and the biological plausibility of synergistic toxicity, future research should explore whether combining PFAS and metal exposures into weighted composite indices may enhance the prediction of cardiovascular outcomes. Although our use of BKMR-CMA enabled a flexible and non-parametric evaluation of joint effects and mediation pathways, it does not produce summary scores or relative importance weights for individual exposures. Developing an importance-weighted exposure index—based on toxicological potency, detection frequency, or statistical contribution—could offer additional interpretability and policy relevance. Such approaches may help quantify mixture burden and identify key contributors to cardiovascular risk. Integrating this with causal frameworks could also improve our ability to design targeted interventions, particularly for populations with high cumulative exposure burdens. Additionally, longitudinal studies are needed to better establish temporal relationships between exposures and cardiovascular outcomes, while mechanistic investigations could elucidate the molecular pathways underlying age-dependent responses. Intervention studies focusing on dietary modification and exposure reduction could provide practical insights for public health applications. Furthermore, methodological developments in mixture analysis and exposure assessment techniques could enhance our understanding of these complex relationships.

## 5. Conclusions

The exploration of the mediating role of diet in the relationship between environmental pollutants and cardiovascular disease reveals a complex interplay that warrants further investigation. While dietary patterns can influence inflammation and overall cardiovascular health, their ability to modify the toxicological effects of PFAS and heavy metals appears limited. Age was identified as an effect modifier, with younger individuals appearing more susceptible to the adverse effects of combined exposure to PFAS and heavy metals on lipid profiles and inflammation. These findings underscore the importance of continued public health interventions, dietary recommendations, and regulatory measures to reduce combined pollution exposure and mitigate cardiovascular disease risk. Future research should aim to clarify the mechanisms underlying these interactions and develop comprehensive strategies that integrate dietary and environmental health considerations to effectively reduce cardiovascular disease risk.

## Supplementary Material

Supplementary materials Figure S1: The Overall exposure effect of combined PFAS and Metals on DBP examined at 0.25–0.75 quantiles of exposure as compared to the 0.5 quantile; Figure S2: Causal Mediation analysis assessing the combined effect of PFAS and Metals on DBP with DII as mediator; Figure S3: Overall exposure effect of combined PFAS and Metals on HDL examined at 0.25–0.75 quantiles of exposure as compared to the 0.5 quantile; Figure S4: Causal Mediation analysis assessing the combined effect of PFAS and Metals on HDL with DII as mediator; Figure S5: Overall exposure effect of combined PFAS and Metals on Total Cholesterol examined at 0.25–0.75 quantiles of exposure as compared to the 0.5 quantile; Figure S6: Causal Mediation analysis assessing the combined effect of PFAS and Metals on Total Cholesterol with DII as mediator; Figure S7: The overall exposure effect of combined PFAS and metals on triglycerides examined at 0.25–0.75 quantiles of exposure compared to the 0.5 quantile; Figure S8: Causal Mediation analysis assessing the combined effect of PFAS and Metals on Triglycerides with DII as mediator; Figure S9: Overall exposure effect of combined PFAS and Metals on CRP examined at 0.25–0.75 quantiles of exposure as compared to the 0.5 quantile; Figure S10: Causal Mediation analysis assessing the combined effect of PFAS and Metals on CRP with DII as mediator; Figure S11: The Overall exposure effect of combined PFAS and Metals on LDL examined at 0.25–0.75 quantiles of exposure as compared to the 0.5 quantile; Figure S12: Causal Mediation analysis assessing the combined effect of PFAS and Metals on LDL with DII as mediator; Table S1: Linear Regression of Association between Diastolic Blood Pressure with PFAS, Metals and DII; Table S2: Linear Regression of Association between High-Density Lipoproteins (HDL) with PFAS, Metals and DII; Table S3: Linear Regression of Association between Triglycerides (TG) with PFAS, Metals, and DII; Table S4: Linear Regression of Association between Total Cholesterol (TC) with PFAS, Metals, and DII; Table S5: Linear Regression of Association between C-Reactive Protein with PFAS, Metals and DII; Table S6: Linear Regression of Association between Low-Density Lipoproteins with PFAS, Metals and DII.

**Supplementary Materials:** The following supporting information can be downloaded at: https://www.mdpi.com/article/10.3390/environments12060178/s1

## Figures and Tables

**Table 1.** Mean levels of critical variables of interest, including age in years, BMI (kg/m^2^), PFAS (PFOA and PFOS [measured in serum]), lead, cadmium, and mercury [measured in whole blood], the DII, and cardiovascular markers.

Variable	Participants (n)	Mean	Standard Deviation (SD)
Age (Years)	660	49.33	18.51
BMI (kg/m^2^)	660	29.80	7.79
Lead (μg/dL)	660	1.21	1.27
Cadmium (μg/L)	660	0.45	0.47
Mercury (μg/L)	660	1.24	1.83
PFOA (ng/mL)	660	1.69	1.21
PFOS (mg/mL)	660	6.94	8.25
DII	660	1.50	1.68
SBP (mm Hg)	660	125.01	19.15
DBP (mm Hg)	660	71.86	12.27
Total Cholesterol (mg/dL)	660	184.00	42.19
Triglycerides (mg/dL)	660	103.32	62.74
C-Reactive Protein (mg/L)	660	4.48	10.65
LDL Cholesterol (mg/dL)	660	109.40	36.73
HDL Cholesterol (mg/dL)	660	53.96	16.31

**Table 2.** Linear regression of association between systolic blood pressure with PFAS, metals, and DII.

SBP	Coefficient	Std. Error	*p*-Value	95% Confidence Interval
Lead (μg/dL)	0.604	0.530	0.255	−0.436, 1.644
Cadmium (μg/L)	−1.541	1.424	0.279	−4.338, 1.255
Mercury (μg/L)	−0.948	0.395	0.017	−1.723, −0.172
PFOA (ng/mL)	1.025	0.612	0.095	−0.178, 0.0934
PFOS (mg/mL)	−0.0845	0.0906	0.352	−0.262, 0.0934
DII	0.223	0.395	0.572	−0.552, 0.100

## Data Availability

The data presented in this study are openly available on the CDC NHANES site at https://wwwn.cdc.gov/nchs/nhanes/continuousnhanes/overview.aspx?BeginYear=2017 (accessed on 1 December 2024).
