# Supplementary material for: Association of PFAS and Metals with Cardiovascular Disease Risk: Exploring the Mediating Effect of Diet
Source: Environments (Basel). Author manuscript; Available in PMC 2025 Aug 21. (PMC12366770; doi:10.3390/environments12060178)
Supplement: Supplementary materials — Figure S1: The Overall exposure effect of combined PFAS and Metals on DBP examined at 0.25–0.75 quantiles of exposure as compared to the 0.5 quantile; Figure S2: Causal Mediation analysis assessing the combined effect of PFAS and Metals on DBP with DII as mediator; Figure S3: Overall exposure effect of combined PFAS and Metals on HDL examined at 0.25–0.75 quantiles of exposure as compared to the 0.5 quantile; Figure S4: Causal Mediation analysis assessing the combined effect of PFAS and Metals on HDL with DII as mediator; Figure S5: Overall exposure effect of combined PFAS and Metals on Total Cholesterol examined at 0.25–0.75 quantiles of exposure as compared to the 0.5 quantile; Figure S6: Causal Mediation analysis assessing the combined effect of PFAS and Metals on Total Cholesterol with DII as mediator; Figure S7: The overall exposure effect of combined PFAS and metals on triglycerides examined at 0.25–0.75 quantiles of exposure compared to the 0.5 quantile; Figure S8: Causal Mediation analysis assessing the combined effect of PFAS and Metals on Triglycerides with DII as mediator; Figure S9: Overall exposure effect of combined PFAS and Metals on CRP examined at 0.25–0.75 quantiles of exposure as compared to the 0.5 quantile; Figure S10: Causal Mediation analysis assessing the combined effect of PFAS and Metals on CRP with DII as mediator; Figure S11: The Overall exposure effect of combined PFAS and Metals on LDL examined at 0.25–0.75 quantiles of exposure as compared to the 0.5 quantile; Figure S12: Causal Mediation analysis assessing the combined effect of PFAS and Metals on LDL with DII as mediator; Table S1: Linear Regression of Association between Diastolic Blood Pressure with PFAS, Metals and DII; Table S2: Linear Regression of Association between High-Density Lipoproteins (HDL) with PFAS, Metals and DII; Table S3: Linear Regression of Association between Triglycerides (TG) with PFAS, Metals, and DII; Table S4: Linear Regression of Association between Total [file NIHMS2092389-supplement-Supplementary_materials.zip › environments-3559535-supplementary.pdf]

environments

MDPI

Association of PFAS and Metals with Cardiovascular Disease Risk: Exploring the Mediating Effect of Diet

Supplementary Materials

1. Linear regression results

The linear regression analysis investigated the relationship between diastolic blood pressure, PFAS, metals, and DII is presented in Supplementary Table 1. Lead (β = 0.555, p = 0.157), cadmium (β = -1.488, p = 0.157), mercury (β = 0.211, p = 0.469), PFOA (β = 0.295, p = 0.515), PFOS (β = -0.068, p = 0.309), and DII (β = -0.270, p = 0.355) all showed non-significant relationships with DBP. These findings suggest that PFAS and metals may have limited direct effects on diastolic blood pressure in this population.

Supplementary Table 1. Linear Regression of Association between Diastolic Blood Pressure with PFAS, Metals and DII.

DBP	Coefficient	Std. error	p-value	95% Confidence Interval
Lead (µg/dL)	0.555	0.391	0.157	-0.213, 1.322
Cadmium (µg/L)	-1.488	1.051	0.157	-3.553, 0.576
Mercury (µg/L)	0.211	0.291	0.469	-0.362, 0.784
PFOA (ng/mL)	0.295	0.452	0.515	-0.593, 1.183
PFOS (mg/mL)	-0.068	0.0668	0.309	-0.199, 0.0633
DII	-0.270	0.292	0.355	-0.843, 0.303

The analysis presented in Supplementary Table 2 highlights the relationship between PFAS, metals, and DII with HDL levels. A statistically significant positive association was observed between mercury exposure (µg/L) and HDL levels (β = 1.296, 95% CI: 0.567–2.025, p = 0.001), suggesting that each unit increase in mercury concentration correlated with a 1.3-unit rise in HDL. Conversely, PFOA demonstrated a statistically significant negative association with HDL (β = -1.323, *p* = 0.022), suggesting that elevated PFOA exposure may lead to reduced HDL levels. Lead (β = 0.734, p = 0.141), cadmium (β = 0.313, p = 0.815), (PFOS, mg/mL; β = 0.053, p = 0.532), and the Dietary Inflammatory Index (DII; β = -0.362, p = 0.330) showed no significant association with HDL.

Supplementary Table 2. Linear Regression of Association between High-Density Lipoproteins (HDL) with PFAS, Metals and DII.

HDL	Coefficient	Std. error	p-value	95% Confidence Interval
Lead (µg/dL)	0.734	0.498	0.141	-0.244, 1.711
Cadmium (µg/L)	0.313	1.339	0.815	-2.316, 2.942
Mercury (µg/L)	1.296	0.371	0.001	0.567, 2.025
PFOA (ng/mL)	-1.323	0.576	0.022	-2.453, -0.192
PFOS (mg/mL)	0.0532	0.0852	0.532	-0.114, 0.220
DII	-0.362	0.371	0.330	-1.091, 0.367

As shown in Supplementary Table 3, we assessed the associations between triglyceride levels and exposure to PFAS, metals, and DII, adjusted for covariates. PFOS exposure exhibited a statistically significant relationship with triglycerides. Lead (coefficient = -1.906, p = 0.329), cadmium (coefficient = -1.955, p = 0.710), mercury (coefficient = 0.137, p = 0.925), and PFOA (coefficient = 3.184, p = 0.159) did not exhibit significant association with triglyceride (TG) levels. DII showed no statistically significant negative relationship with TG levels (coefficient = -2.284, p = 0.117).

Supplementary Table 3. Linear Regression of Association between Triglycerides (TG) with PFAS, Metals, and DII.

TG	Coefficient	Std. error	p-value	95% Confidence Interval
Lead (µg/dL)	-1.906	1.952	0.329	-5.739, 1.927
Cadmium (µg/L)	-1.955	5.250	0.710	-12.264, 8.354
Mercury (µg/L)	0.1370	1.456	0.925	-2.722, 2.996
PFOA (ng/mL)	3.184	2.257	0.159	-1.249, 7.617
PFOS (mg/mL)	-0.821	0.334	0.014	-1.476, -0.165
DII	-2.284	1.456	0.117	-5.14, 0.576

The linear relationship between total cholesterol and PFAS, metals, and DII were assessed, as illustrated in Supplementary Table 4. Our analysis revealed that mercury (coefficient = 1.886, p = 0.060) has a marginally

significant positive association with total cholesterol (TC), suggesting that mercury level may influence overall cholesterol metabolism.

Lead (coefficient = 1.726, p = 0.199) and cadmium (coefficient = 3.298, p = 0.362) also showed no statistically significant positive associations with TC. Similarly, PFOA (coefficient = -0.898, p = 0.563) and PFOS (coefficient = 0.0610, p = 0.791) exhibited no significant negative associations with TC. The Dietary Inflammatory Index (DII) was negatively associated with TC (coefficient = -1.124, p = 0.262), but this relationship was not statistically significant

Supplementary Table 4. Linear Regression of Association between Total Cholesterol (TC) with PFAS, Metals, and DII.

TC	Coefficient	Std. error	p-value	95% Confidence Interval
Lead (μg/dL)	1.726	1.343	0.199	-0.911, 4.364
Cadmium (μg/L)	3.298	3.613	0.362	-3.796, 10.393
Mercury (μg/L)	1.886	1.0019	0.060	-0.0814, 3.853
PFOA (ng/mL)	-0.898	1.554	0.563	-3.949, 2.153
PFOS (mg/mL)	0.0610	0.230	0.791	-0.390, 0.512
DII	-1.124	1.0021	0.262	-3.092, 0.843

As summarized in Supplementary table 5, Cadmium revealed a statistically significant positive relationship with CRP (coefficient = 2.434, p = 0.006), suggesting a potential inflammatory response to cadmium exposure. The dietary inflammatory index (DII) also showed a marginally significant positive relationship with CRP (coefficient = 0.445, p = 0.069), indicating that dietary patterns may influence systemic inflammation. Lead, mercury, PFOA, and PFOS demonstrated no significant association with CRP levels.

Supplementary Table 5. Linear Regression of Association between C-Reactive Protein with PFAS, Metals and DII.

CRP	Coefficient	Std. error	p-value	95% Confidence Interval
Lead (μg/dL)	0.123	0.328	0.708	-0.521, 0.767
Cadmium (μg/L)	2.434	0.882	0.006	0.701, 4.166

CRP	Coefficient	Std. error	p-value	95% Confidence Interval
Mercury (μg/L)	-0.0840	0.245	0.732	-0.564, 0.396
PFOA (ng/mL)	-0.502	0.379	0.186	-1.247, 0.243
PFOS (mg/mL)	-0.0407	0.056	0.469	-0.151, 0.0695
DII	0.445	0.245	0.069	-0.0355, 0.926

Supplementary Table 6 examined the associations between low-density lipoprotein (LDL) levels, PFAS, metals, and DII. Lead (β = 1.366, *p* = 0.247) and cadmium (β = 3.364, *p* = 0.289) exhibited positive coefficients, exhibited positive coefficients, though the relationship was not significant. Similarly, mercury (β = 0.564, p = 0.521), perfluorooctanoic acid (PFOA; β = -0.234, p = 0.863), and PFOS (β = 0.175, p = 0.386) showed no significant relationships with LDL. The Dietary Inflammatory Index (DII) also revealed no significant association (β = -0.310, p = 0.724).

Supplementary Table 6. Linear Regression of Association between Low-Density Lipoproteins with PFAS, Metals and DII.

LDL	Coefficient	Std. error	p-value	95% Confidence Interval
Lead (μg/dL)	1.366	1.178	0.247	-0.947, 3.679
Cadmium (μg/L)	3.364	3.168	0.289	-2.857, 9.585
Mercury (μg/L)	0.564	0.876	0.521	-1.161, 2.289
PFOA (ng/mL)	-0.234	1.632	0.863	-2.910, 2.440
PFOS (mg/mL)	0.175	0.202	0.386	-0.221, 0.571
DII	-0.310	0.879	0.724	-2.036, 1.415

2. BKMR Results

The combined effects of PFAS and metals on diastolic blood pressure, as analyzed using BKMR, revealed the following (Supplementary Figure 1):

Left Plot: 10th Percentile of Age (Younger Age Group)

- Effect Estimates**:** The effect estimates (est) hover around zero across the quantiles, with slight fluctuations.

- Trend: There is a slight increasing trend across the exposure quantiles for younger individuals, as the effect estimates remain close to zero or slightly negative at lower quantiles and slightly positive at higher quantiles.
- Credible Intervals: The credible intervals are relatively wide, especially at higher quantiles, indicating uncertainty in the estimates. This suggests that PFAS and metals have minimal and uncertain effects on DBP for younger individuals.

Right Plot: 90th Percentile of Age (Older Age Group)

- Effect Estimates: For older individuals, the effect estimates are again close to zero across most quantiles, with a slight upward trend at higher quantiles.
- Trend: There is a mild increase in estimated effect at higher quantiles, suggesting that for older individuals, high exposure levels might be associated with a slight increase in DBP, though the effect remains small.
- Credible Intervals: The credible intervals are wide, particularly at extreme quantiles, showing a high degree of uncertainty. This makes it difficult to confirm any significant association between PFAS/metals exposure and DBP in this age group.

Summary - Overall, the chart demonstrates PFAS and metals' impact on DBP for both younger and older individuals. There is a slight tendency for DBP to increase at higher exposure quantiles for older individuals, but the effect is uncertain due to wide credible intervals.

**DBP Risk summary for 10th percentile age effect modifier**	**DBP Risk summary for 90th percentile age effect modifier**
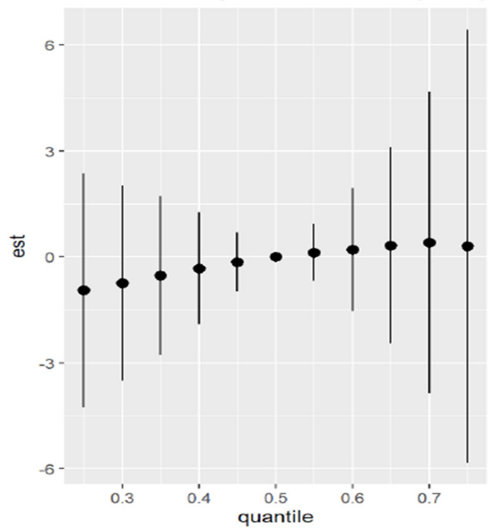	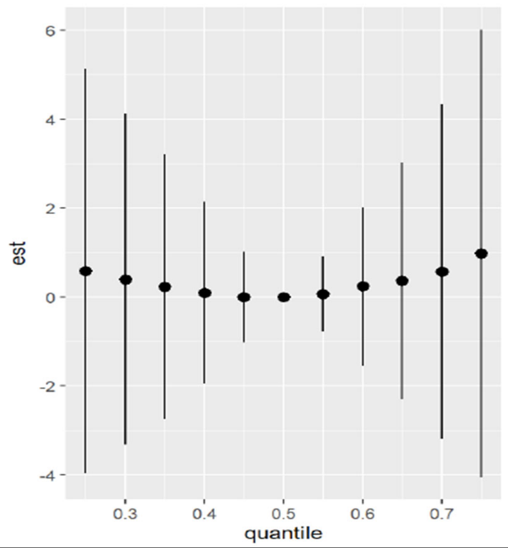

Supplementary Figure 1. The Overall exposure effect of combined PFAS and Metals on DBP examined at 0.25 – 0.75 quantiles of exposure as compared to the 0.5 quantile.

BKMR-CMA was also used to estimate the direct and indirect effects of PFAS and Metals mixtures on DBP through the DII level according to age (Supplementary Figure 2). The results were as follows:

Left Plot: 10th Percentile of Age (Younger Age Group)

- TE (Total Effect): The total effect, which includes both direct and indirect effects, is close to zero, and the credible interval is large and crosses zero, indicating substantial uncertainty and minimal overall impact of PFAS and metals on DBP in this age group.
- NDE (Natural Direct Effect): The direct effect, independent of DII, is also close to zero, with a large credible interval crossing zero, suggesting that PFAS and metals do not have a substantial direct effect on DBP in younger individuals, though the estimate remains highly uncertain.
- NIE (Natural Indirect Effect): This represents the portion of the exposure effect on DBP that is mediated through the Dietary Inflammatory Index (DII). The estimate is close to zero, and the large credible interval crosses zero, indicating significant uncertainty.
- CDEs at Different Quantiles (10%, 25%, 50%, 75%): The Controlled Direct Effects show a slight downward trend across the DII quantiles, where the effect of PFAS and metals exposure on DBP levels becomes marginally more negative. However, all credible intervals are large and cross zero, suggesting that the results remain highly uncertain and should be interpreted cautiously.

Right Plot: 90th Percentile of Age (Older Age Group)

- TE (Total Effect): The total effect is close to zero, and the credible interval is large and crosses zero, suggesting substantial uncertainty and no clear evidence that PFAS and heavy metals exposure directly impact DBP in the older age group.
- NDE (Natural Direct Effect): For older adults, the NDE is weak, with a sizeable credible interval crossing zero, indicating a weak or negligible direct association with DBP and considerable uncertainty in the estimate.
- NIE (Natural Indirect Effect): The NIE is negative but close to zero, and the sizeable credible interval crosses zero, suggesting significant uncertainty.
- CDEs at Different Quantiles of DII (10%, 50%, 75%): The Controlled Direct Effects show a slight upward trend, suggesting that the effect of PFAS and metals exposure on DBP levels becomes marginally more positive as DII quantiles increase. However, all credible intervals are large and cross zero, indicating that while there may be

a weak positive association, the results remain highly uncertain and should be interpreted cautiously.

Summary - Across both age groups, the results suggest that PFAS and metals have a minimal and highly uncertain impact on DBP, as all credible intervals are large and cross zero, indicating substantial uncertainty in the estimated effects. No clear direct, indirect, or total effects were observed. Additionally, DII does not appear to mediate this relationship, and controlling for different levels of DII does not meaningfully alter the estimated effects of PFAS/metals on DBP. This implies that, within the scope of this analysis, age (as an effect modifier) and dietary inflammation (DII as a mediator) do not play a substantial role in shaping the relationship between PFAS/metals exposure and DBP, though the wide credible intervals suggest these findings should be interpreted with caution.

**DBP Risk summary for 10th percentile age effect modifier**	**DBP Risk summary for 90th percentile effect age modifier**
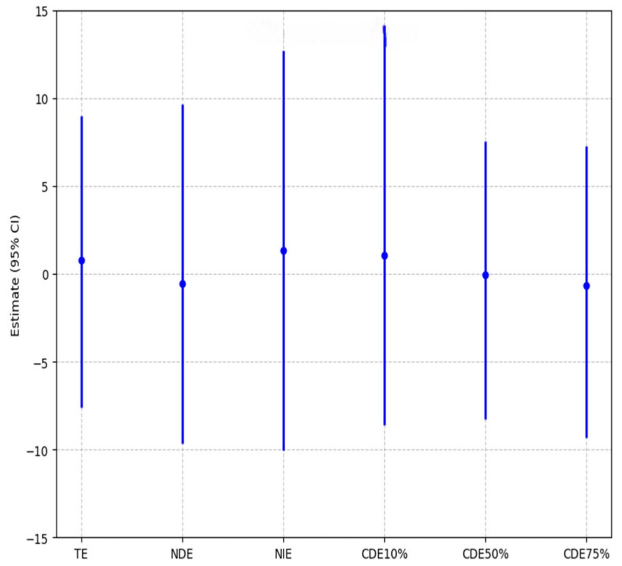	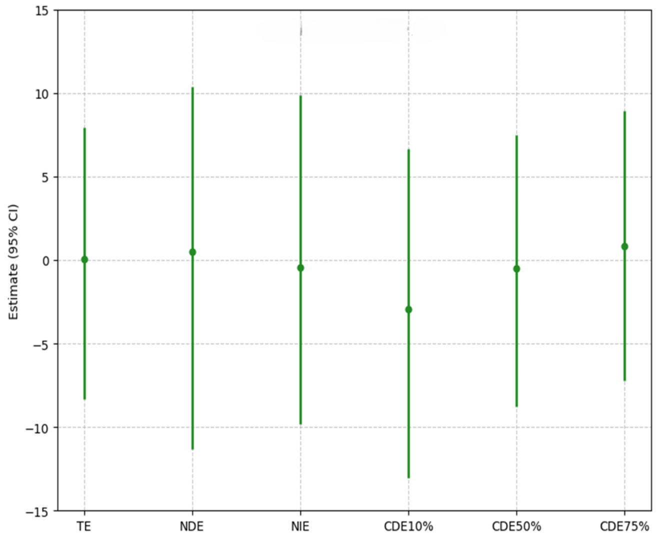

Supplementary Figure 2. Causal Mediation analysis assessing the combined effect of PFAS and Metals on DBP with DII as mediator.

HDL (High-Density Lipoprotein) Cholesterol

The combined effects of PFAS and metals on HDL, as analyzed using BKMR, revealed the following (Supplementary Figure 3):

The chart displays the HDL (High-Density Lipoprotein) Risk Summary for different quantiles of PFAS and metals exposure, with age as an effect modifier at the 10th and 90th percentiles. The estimated effect (est) and credible intervals are shown for each quantile of exposure.

Left Plot: 10th Percentile of Age (Younger Age Group)

- Effect Estimates: The effect estimates show a decreasing trend as exposure quantiles increase, moving from slightly positive values to negative values at higher quantiles.
- Trend: This decreasing trend suggests that in younger individuals, higher levels of PFAS and metals exposure may be associated with a decrease in HDL levels, though the effect is not statistically significant.
- Credible Intervals: The credible intervals are quite wide, particularly at the higher quantiles, which indicates greater uncertainty in the effect estimates at higher exposure levels. Despite this uncertainty, the downward trend implies a potential negative association between exposure and HDL in younger individuals.

Right Plot: 90th Percentile of Age (Older Age Group)

- Effect Estimates: For older individuals, the effect estimates show a positive trend across all exposure quantiles.
- Trend: There is a slight upward trend across quantiles for older individuals, suggesting that PFAS and metals exposure have a slight positive relationship with HDL levels in this age group.
- Credible Intervals: The credible intervals are also wide but tend to narrow slightly at higher quantiles, indicating high uncertainty in the estimates. However, the estimates still overlap zero at all quantiles, implying uncertainty in the relationship between exposure and HDL in older adults.

Summary - The results suggest an age-dependent effect of PFAS and metals on HDL levels:

- Younger Age Group (10th Percentile): There appears to be a negative association between PFAS and metals exposure and HDL levels, particularly at higher exposure quantiles. However, the wide credible intervals crossing zero indicate substantial uncertainty, meaning that while the trend suggests a possible decrease in HDL levels with higher exposure, this relationship should be interpreted with caution.

- Older Age Group (90th Percentile): There appears to be a modest positive association between PFAS and metals exposure and HDL levels, particularly at higher exposure quantiles. However, the wide credible intervals crossing zero indicate significant uncertainty, suggesting that while the trend may indicate a potential increase in HDL levels with higher exposure, this relationship should be interpreted with caution.

These results indicate a potential interaction between age and PFAS/metals exposure on HDL, where younger individuals may experience a slight

decrease in HDL with higher exposure levels, while older individuals show no significant association. However, the wide credible intervals suggest that these findings should be interpreted with caution.

**HDL Risk summary for 10th percentile age effect modifier**	**HDL Risk summary for 90th percentile age effect modifier**
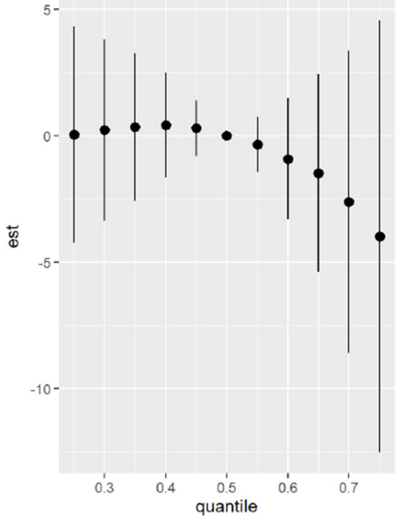	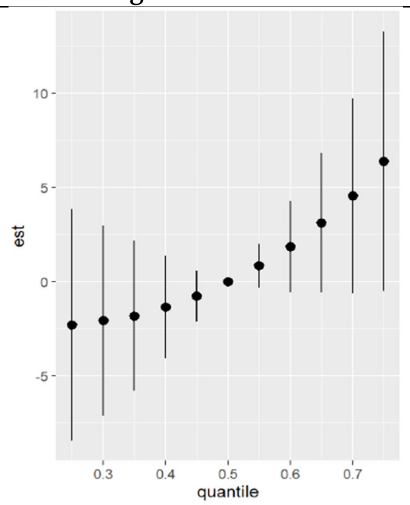

Supplementary Figure 3. Overall exposure effect of combined PFAS and Metals on HDL examined at 0.25 – 0.75 quantiles of exposure as compared to the 0.5 quantile.

BKMR-CMA was also used to estimate the direct and indirect effects of PFAS and Metals mixtures on HDL through the DII level according to age (Supplementary Figure 4). The results were as follows:

This plot presents the Causal Mediation Analysis results for HDL with PFAS and metals as exposures, using age as an effect modifier at the 10th and 90th percentiles. Each panel shows the estimates for different types of effects: Natural Indirect Effect (NIE)**,** Natural Direct Effect (NDE)**,** Total Effect (TE)**,** and Controlled Direct Effects at different quantiles of the mediator (likely the Dietary Inflammatory Index, DII) with 95% credible intervals.

Left Plot: 10th Percentile of Age (Younger Age Group)

- TE (Total Effect): The total effect, combining both direct and indirect effects, suggests that combined PFAS/metals exposure **may** be associated with a slight decrease in HDL in this age group. However, the wide credible intervals crossing zero indicate substantial uncertainty.
- NDE (Natural Direct Effect): The NDE is negative, suggesting that a slight decrease in HDL could be due to combined PFAS/metals exposure in younger individuals when DII is controlled for. However, the large credible interval crossing zero indicates high uncertainty, making this effect inconclusive.
- NIE (Natural Indirect Effect): The NIE is slightly positive, implying that DII may slightly mediate the relationship between PFAS/metals

exposure and HDL for younger individuals, with higher DII potentially resulting in higher HDL. However, the wide credible intervals crossing zero indicate that this mediation effect is highly uncertain.

- CDEs at Different Quantiles of DII (10%, 50%, 75%): As Controlled Direct Effects (CDEs) increase, the effect on HDL appears to increase. However, all credible intervals are large and cross zero, highlighting significant uncertainty in this relationship, and the results should be interpreted with caution.

Right Plot: 90th Percentile of Age (Older Age Group)

- TE (Total Effect): The total effect in older adults is slightly positive, suggesting that combined PFAS and metals exposure may be associated with a slight increase in HDL. However, the wide credible intervals crossing zero indicate substantial uncertainty.
- NDE (Natural Direct Effect): In contrast to the younger age group, the NDE is slightly positive, suggesting that PFAS/metals may be associated with a slight increase in HDL in older individuals. However, the large credible interval crossing zero indicates high uncertainty, making this effect inconclusive.
- NIE (Natural Indirect Effect): The NIE is slightly negative, suggesting that lower DII may be associated with higher HDL in older individuals. However, the wide credible intervals crossing zero indicate that this mediation effect is highly uncertain, and the results should be interpreted cautiously.
- CDEs at Different Quantiles of DII (10%, 50%, 75%): Controlled Direct Effects (CDEs) are slightly negative across all levels of DII. However, all credible intervals are large and cross zero, highlighting significant uncertainty.

Summary

The results suggest that PFAS and metals exposure **may** have varying effects on HDL across both younger and older age groups, with both direct and indirect effects appearing slightly more pronounced in younger individuals. However, the wide credible intervals crossing zero indicate substantial uncertainty in these estimates. Younger individuals show a potential slight negative association, while older individuals appear to have a slight positive association. Given the high degree of uncertainty, these findings should be interpreted with caution.

**HDL Risk summary for 10th percentile age effect modifier**	**HDL Risk summary for 90th percentile age effect modifier**

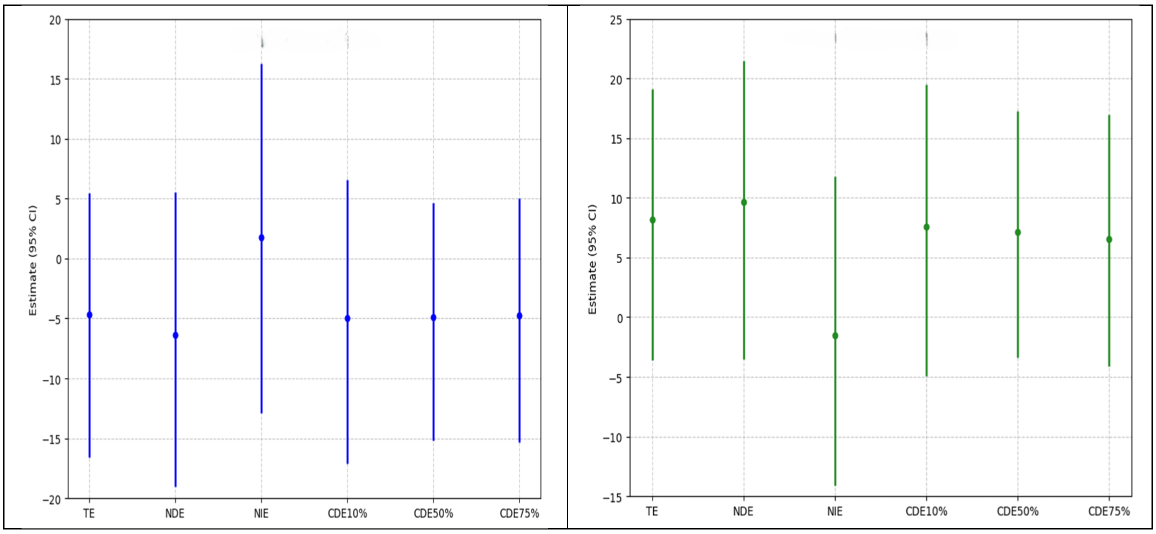

Supplementary Figure 4. Causal Mediation analysis assessing the combined effect of PFAS and Metals on HDL with DII as mediator.

4.4.6 Total Cholesterol

The combined effects of PFAS and metals on Total Cholesterol, as analyzed using BKMR, revealed the following (Supplementary Figure 5):

Left Plot: 10th Percentile of Age (Younger Age Group)

- Effect Estimates: The effect estimates begin with negative values at lower quantiles and then trend upwards, becoming positive at higher quantiles. However, the wide credible intervals crossing zero suggest considerable uncertainty in the estimates, preventing strong conclusions about this trend.
- Trend: This apparent upward trend suggests that lower levels of PFAS and metal exposure may be associated with a slight decrease in total cholesterol in younger individuals. However, as exposure levels increase, the association appears to become positive, indicating a potential increase in total cholesterol until a threshold is met, after which the effect seems to decline. Given the wide credible intervals, particularly at higher quantiles, this pattern remains highly uncertain.

- Credible Intervals: The credible intervals are wide across all quantiles and particularly large at higher exposure levels, indicating greater uncertainty in the effect estimates. While an upward trend is observed, the large credible intervals crossing zero suggest that this apparent positive association between PFAS/metals exposure and total cholesterol at higher exposure quantiles is highly uncertain and should be interpreted cautiously.

Right Plot: 90th Percentile of Age (Older Age Group)

- Effect Estimates: For older individuals, the effect estimates remain close to zero across all quantiles, with no clear upward or downward trend. This indicates that PFAS and metals exposure does not appear to have a meaningful association with total cholesterol in this age group.
- Trend: Unlike younger individuals, no noticeable pattern emerges in older individuals, as effect estimates fluctuate around zero across all exposure levels. This suggests that PFAS and metals exposure does not show a clear relationship with total cholesterol in this age group.
- Credible Intervals: The wide credible intervals consistently crossing zero across all quantiles indicate substantial uncertainty in the estimates.

Summary

While the younger age group shows some variability in total cholesterol response to PFAS/metals exposure at different quantiles, the results for older individuals do not indicate a strong pattern. In both groups, credible intervals are wide and cross zero at all quantiles, meaning there is no strong statistical support for a dose-response relationship between PFAS/metals exposure and total cholesterol. These findings should be interpreted with caution due to the high degree of uncertainty.

**Total Cholesterol Risk summary for 10th percentile age effect modifier**	**Total Cholesterol Risk summary for 90th percentile age effect modifier**
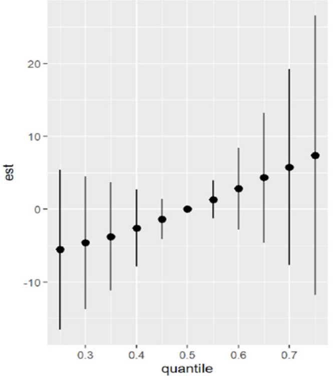	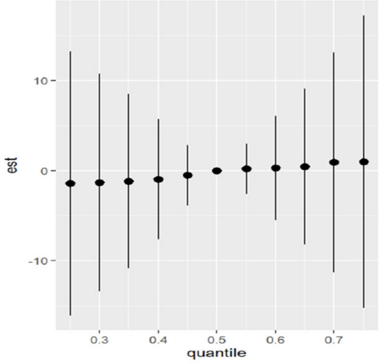

Supplementary Figure 5. Overall exposure effect of combined PFAS and Metals on Total Cholesterol examined at 0.25 – 0.75 quantiles of exposure as compared to the 0.5 quantile.

BKMR-CMA was also used to estimate the direct and indirect effects of PFAS and Metals mixtures on Total Cholesterol through the DII level according to age (Supplementary Figure 6). The results were as follows:

Left Plot: 10th Percentile of Age (Younger Age Group)

- TE (Total Effect): The total effect is positive, suggesting that PFAS/metals exposure may be associated with a slight increase in total cholesterol. However, the wide credible intervals crossing zero indicate considerable uncertainty.
- NDE (Natural Direct Effect): The NDE is negative, suggesting a minimal direct decrease in total cholesterol due to exposure to PFAS and heavy metals when DII is controlled for. However, the large credible interval crossing zero indicates substantial uncertainty, making this effect inconclusive.
- NIE (Natural Indirect Effect): The NIE is positive, suggesting that DII may mediate a slight increase in total cholesterol due to PFAS and metals exposure in younger individuals. However, since the credible interval is large and includes zero, this mediation effect is highly uncertain.
- CDEs at Different Quantiles of DII (10%, 50%, 75%): The Controlled Direct Effects remain close to zero across all quantiles, implying that controlling for different levels of DII does not reveal any strong direct effect of PFAS/metals exposure on total cholesterol in younger individuals. The wide credible intervals crossing zero further highlight the lack of a clear association.

Right Plot: 90th Percentile of Age (Older Age Group)

- TE (Total Effect): The total effect is close to zero, suggesting that combined exposure to PFAS and heavy metals is not strongly or consistently associated with total cholesterol in older individuals. However, the wide credible interval crossing zero indicates substantial uncertainty, meaning this result should be interpreted cautiously.
- NDE (Natural Direct Effect): The NDE is positive and slightly above 20, suggesting a potential direct increase in total cholesterol due to PFAS and heavy metals exposure. However, the large credible interval crossing zero indicates significant uncertainty, preventing strong conclusions about this direct effect.
- NIE (Natural Indirect Effect): The NIE is negative, suggesting that DII may mediate a modest decrease in total cholesterol due to PFAS and heavy metals exposure. However, since the credible interval includes zero, this mediation effect is highly uncertain and not strongly supported by the data.

- CDEs at Different Quantiles of DII (10%, 50%, 75%): The Controlled Direct Effects are negative and relatively close to zero, indicating that the direct effects of PFAS/metals exposure on total cholesterol remain minimal across all levels of DII. However, the wide credible intervals crossing zero indicate high uncertainty.

Summary

The results do not provide strong evidence that the association between PFAS and metals exposure and total cholesterol differs meaningfully by age, as all credible intervals are wide and cross zero, indicating substantial uncertainty. While there is a slight indication that PFAS and metals exposure may minimally increase total cholesterol in younger individuals, and DII may mediate this effect, the high degree of uncertainty prevents strong conclusions. In older individuals, effect estimates fluctuate around zero, further reinforcing the lack of a clear relationship.

**Total Cholesterol Risk summary for 10th percentile age effect modifier**	**Total Cholesterol Risk summary for 90th percentile age effect modifier**
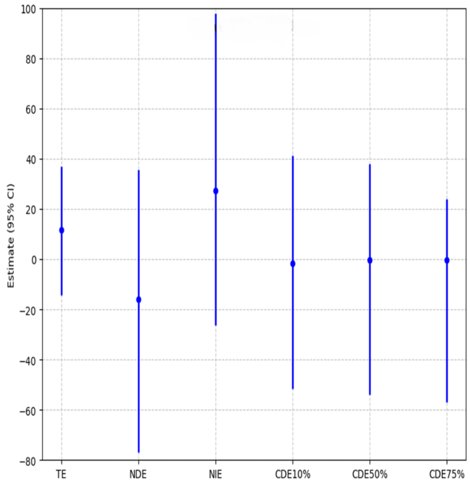	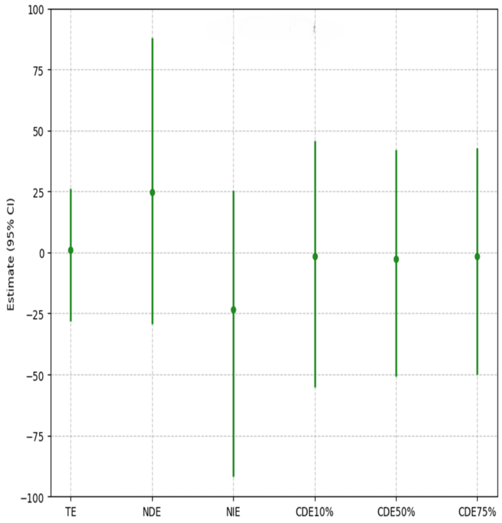

Supplementary Figure 6. Causal Mediation analysis assessing the combined effect of PFAS and Metals on Total Cholesterol with DII as mediator.

4.4.7 Triglycerides

The combined effects of PFAS and metals on Triglycerides, as analyzed using BKMR, revealed the following (Supplementary Figure 7):

- Younger Age Group (10th Percentile): The relationship between PFAS and metals exposure and triglycerides appears flat, with no clear trend observed across exposure levels. The wide credible intervals crossing zero suggest high uncertainty, meaning there is no strong evidence of an association.
- Older Age Group (90th Percentile): An inverse association appears, suggesting that higher exposure levels may be linked to lower

triglyceride levels. This effect seems to become more pronounced at higher exposure quantiles. However, the wide credible intervals crossing zero indicate substantial uncertainty, making this trend inconclusive.

Summary

While there appears to be an age-specific response where older individuals may experience lower triglyceride levels with increased exposure, the high uncertainty in the estimates prevents strong conclusions. The findings should be interpreted cautiously, and further research is needed to confirm potential age-related differences.

**Triglycerides Risk summary for 10th percentile age effect modifier**	**Triglycerides Risk summary for 90th percentile age effect modifier**
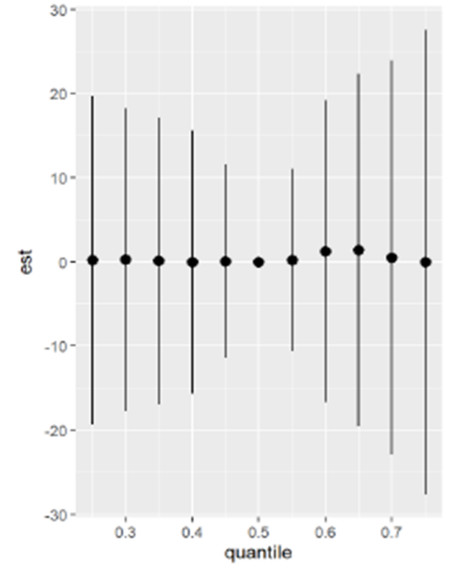	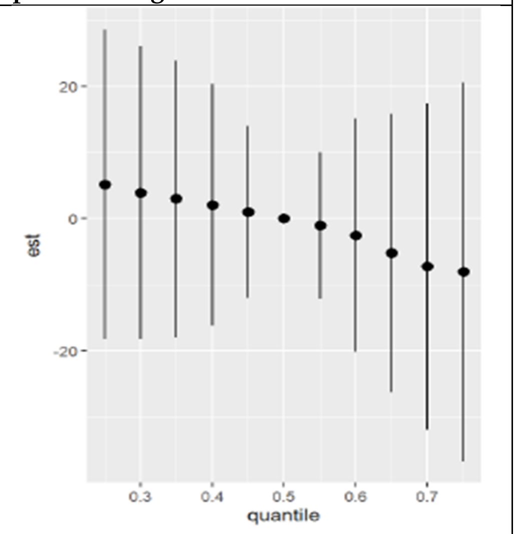

Supplementary Figure 7. The overall exposure effect of combined PFAS and metals on triglycerides examined at 0.25 – 0.75 quantiles of exposure compared to the 0.5 quantile.

BKMR-CMA was also used to estimate the direct and indirect effects of PFAS and Metals mixtures on triglycerides through the DII level according to age (Supplementary Figure 8). The results were as follows:

Left Plot: 10th Percentile of Age (Younger Age Group)

- TE (Total Effect): The total effect is slightly negative, suggesting a potential negative association between PFAS/metals exposure and triglyceride levels. However, the wide credible intervals crossing zero indicate considerable uncertainty, making this effect inconclusive.
- NDE (Natural Direct Effect): The NDE is slightly negative, implying a minimal direct association between PFAS/metals exposure and

triglycerides when DII is controlled for. However, the large credible interval crossing zero suggests that this effect is highly uncertain.

- NIE (Natural Indirect Effect): The NIE is close to zero, suggesting that DII either slightly mediates or does not meaningfully mediate the relationship between PFAS/metals exposure and triglycerides. The wide credible interval crossing zero reinforces the uncertainty of this finding.

Right Plot: 90th Percentile of Age (Older Age Group)

- TE (Total Effect): The total effect is negative, suggesting a potential decrease in triglyceride levels due to PFAS/metals exposure in older individuals. However, the wide credible intervals crossing zero indicate considerable uncertainty, making this effect inconclusive.
- NDE (Natural Direct Effect): The NDE is positive, suggesting a potential direct association between PFAS/metals exposure and triglyceride levels in older individuals. However, the large credible interval crossing zero suggests that this effect is highly uncertain and not well-supported by the data.
- NIE (Natural Indirect Effect): The NIE is slightly negative, suggesting that DII may mediate a small decrease in triglyceride levels due to PFAS/metals exposure. However, the credible interval includes zero, indicating substantial uncertainty and this effect should be interpreted cautiously.
- CDEs at Different Quantiles of DII (10%, 50%, 75%): The Controlled Direct Effects are negative, suggesting that different levels of dietary inflammation do not meaningfully alter the relationship between PFAS/metals exposure and triglycerides in older individuals. However, all credible intervals crossing zero indicate that these effects are uncertain and should not be overinterpreted.

Summary

In younger individuals (10th percentile of age), the Natural Indirect Effect is slightly above zero, suggesting that DII has little to no mediating role in the relationship between PFAS/metals exposure and triglycerides. The Natural Direct Effect and Total Effect are slightly negative, indicating a weak association between exposure and lower triglyceride levels, though wide credible intervals crossing zero suggest high uncertainty. The Controlled Direct Effects remain positive across DII quantiles, but large credible intervals crossing zero indicate significant uncertainty.
In older individuals (90th percentile of age), the NIE is slightly negative, suggesting that DII may have a minor influence in mediating a decrease in triglyceride levels, though with high uncertainty. The NDE is positive, indicating a potential direct association between PFAS/metals exposure and increased triglycerides, while the TE is negative, suggesting an overall reduction in triglycerides. However, wide credible intervals crossing zero prevent strong conclusions. The CDEs remain negative across DII quantiles, suggesting that dietary inflammation does not

meaningfully modify the relationship between PFAS/metals exposure and triglycerides in older individuals.

Triglycerides Risk summary for 10th percentile age effect modifiers	Triglycerides Risk summary for 90th percentile age effect modifiers

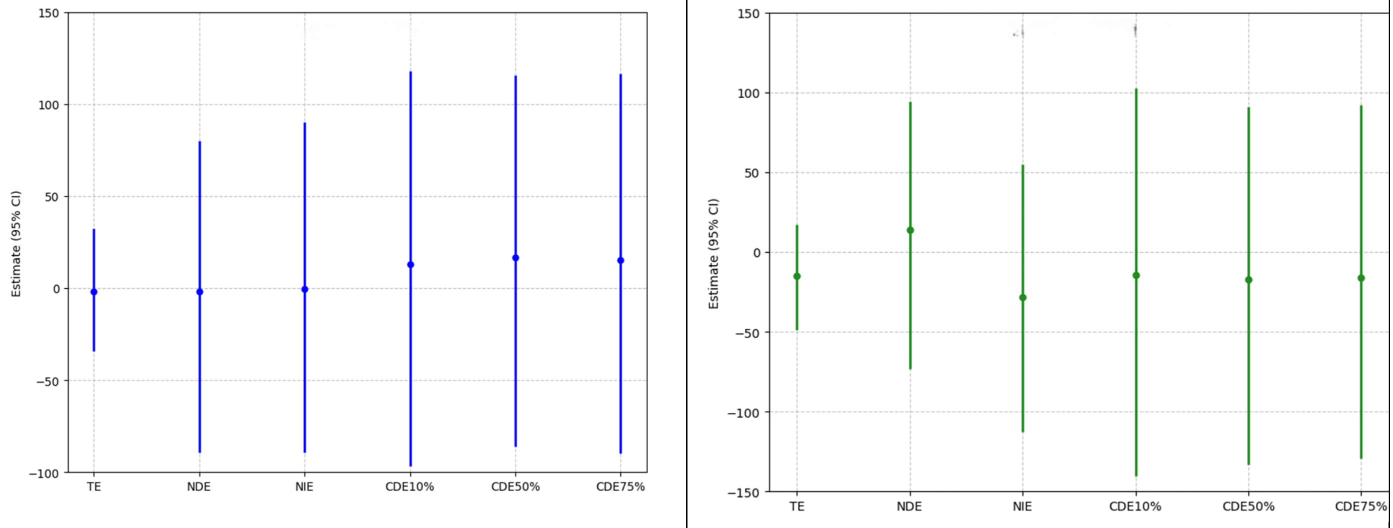

Supplementary Figure 8. Causal Mediation analysis assessing the combined effect of PFAS and Metals on Triglycerides with DII as mediator.

4.4.3 C-reactive protein

The combined effects of PFAS and metals on CRP, as analyzed using BKMR, revealed the following (Supplementary Figure 9):

The chart displays the CRP risk summary for different quantiles of PFAS and metals exposure, with age as an effect modifier at the 10th percentile (younger individuals) and the 90th percentile (older individuals). The estimated effect (est) and credible intervals are shown for each quantile.

Left Plot: 10th Percentile of Age (Younger Age Group)

- Effect Estimates**:** The effect estimates show an upward trend which flattens and remain at zero as exposure quantiles increase, indicating no significant association between PFAS and heavy metals and CRP.
- Trend**:** The flattened pattern suggests that higher levels of exposure to PFAS and metals are not associated with CRP levels for younger individuals.
- Credible Intervals**:** The credible intervals are wide and consistently overlap zero, indicating greater uncertainty and no significant association with exposure and CRP.

Right Plot: 90th Percentile of Age (Older Age Group)

- Effect Estimates: The effect estimates are closer to zero across all exposure quantiles for older individuals.
- Trend: There is no strong pattern across quantiles for older individuals, suggesting that PFAS and metal exposure do not significantly impact CRP levels in this age group.
- Credible Intervals: The credible intervals are wide, especially at the extremes, indicating substantial uncertainty in the estimates and making it difficult to discern a significant association.

Summary – There is a subtle difference between younger and older individuals, suggesting a potential age-dependent variability in CRP to combined PFAS and heavy metals. However, due to wide credible intervals that underscore considerable uncertainty, highlighting the need for cautious interpretation and further investigation.

- Younger Age Group (10th Percentile): Higher PFAS and metal exposure is associated with flat levels of CRP, indicating a potentially minimal effects in younger individuals.
- Older Age Group (90th Percentile): There is minimal association between PFAS/metals exposure and CRP levels, as the effect estimates are close to zero across quantiles, suggesting that exposure does not significantly impact CRP in older individuals.

These findings suggested possible age-related differences in how PFAS and heavy metal exposures affect CRP levels; these observed effects were minimal and not statistically significant, with credible intervals consistently crossing zero.

**CRP Risk summary for 10th percentile age effect modifier**	**CRP Risk summary for 90th percentile age effect modifier**
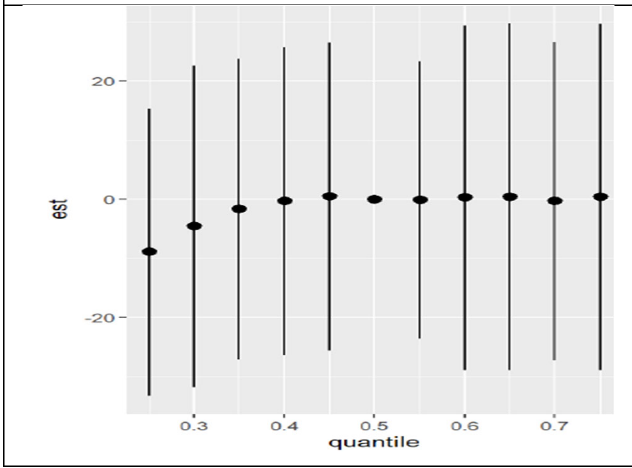	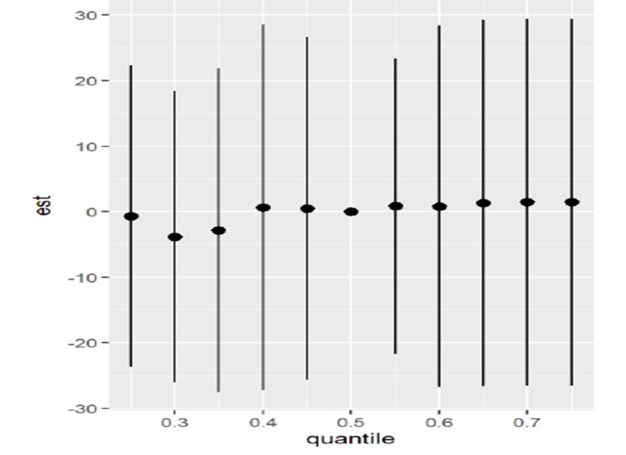

Supplementary Figure 9. Overall exposure effect of combined PFAS and Metals on CRP examined at 0.25 – 0.75 quantiles of exposure as compared to the 0.5 quantile.

BKMR-CMA was also used to estimate the direct and indirect effects of PFAS and Metals mixtures on CRP through the DII level according to age (Supplementary Figure 10). The results were as follows:

Left Plot: 10th Percentile of Age (Younger Age Group)

- TE (Total Effect): The total effect (TE) is close to 10, representing the combined effect of both direct and indirect pathways. However, the wide credible interval crosses zero, indicating substantial uncertainty that higher PFAS and metals exposure **are** associated with increased CRP levels in younger individuals.
- NDE (Natural Direct Effect): The natural direct effect represents the effect of combined PFAS and heavy metals exposure on CRP that is not mediated by DII. The NDE is nearly 10, indicating a slight direct effect of PFAS and metals on increasing CRP in younger individuals, independent of DII. However, the large credible interval crossing zero indicates high uncertainty, meaning that the presence of a direct effect remains inconclusive.
- NIE (Natural Indirect Effect): The natural indirect effect represents the effect of combined PFAS and heavy metals on CRP that is mediated through DII. The NIE is -0.55, suggesting that the Dietary Inflammatory Index (DII) has a negligible effect on CRP due to combined exposure to PFAS and heavy metals. However, the wide credible interval crossing zero suggests that this effect is highly uncertain.
- CDEs at Different Quantiles of DII (10%, 50%, 75%): As the direct effect of PFAS and metals increases, the effect on CRP decreases, with lower direct effects showing a positive relationship with DII in younger individuals. However, all credible intervals are large and cross zero, indicating significant uncertainty in these estimates, and the observed trends should be interpreted with caution.

Right Plot: 90th Percentile of Age (Older Age Group)

NIE, NDE, TE, and CDEs: All effects are close to zero for the older age group, and the wide credible intervals crossing zero indicate substantial uncertainty in these estimates. This suggests that PFAS and metals exposure may have minimal or no clear impact on CRP levels in older individuals. Given the high uncertainty, there is no strong evidence that PFAS and metals significantly affect CRP levels in this age group, regardless of dietary inflammation levels.

Summary - In summary, these findings indicate that:

- In Younger Individuals (10th Percentile of Age): PFAS and metals exposure may have a slight effect on CRP, both directly and indirectly via dietary inflammation (DII), but the wide credible intervals crossing zero indicate substantial uncertainty, meaning this effect is not clearly supported by the data.

- In Older Individuals (90th Percentile of Age): There appears to be minimal or no clear impact of PFAS and metals on CRP, as all credible intervals are large and cross zero, highlighting significant uncertainty. While these results might suggest that younger individuals are more sensitive to PFAS/metals exposure in relation to CRP, the high degree of uncertainty prevents strong conclusions regarding age as an effect modifier.

**CRP Risk summary for 10th percentile age effect modifier**	**CRP Risk summary for 90th percentile age effect modifier**
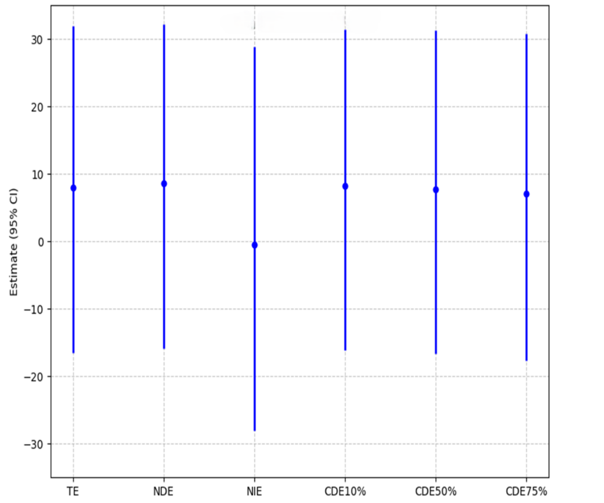	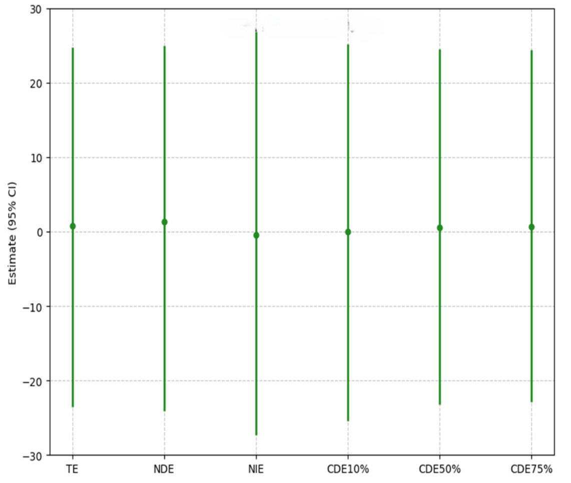

Supplementary Figure 10. Causal Mediation analysis assessing the combined effect of PFAS and Metals on CRP with DII as mediator.

4.4.5 LDL Cholesterol

The combined effects of PFAS and metals on LDL, as analyzed using BKMR, revealed the following (Supplementary Figure 11):

Left Plot: 10th Percentile of Age (Younger Age Group)

- Effect Estimates: The effect estimates across quantiles are consistently negative and fluctuate around zero. However, the wide credible intervals crossing zero indicate substantial uncertainty.
- Trend: This suggests that there may be a minimal association between PFAS/heavy metals exposure and LDL levels for younger individuals. However, this relationship remains highly uncertain, given the large credible intervals and their overlap with zero.
- Credible Intervals: The credible intervals are wide across all quantiles and consistently overlap zero, indicating a high degree of uncertainty.

Right Plot: 90th Percentile of Age (Older Age Group)

- Effect Estimates: For older individuals, the pattern differs from that of the younger group, with estimates starting around zero or slightly positive at lower quantiles and trending downward toward negative values at higher quantiles. **However,** the wide credible intervals crossing zero indicate substantial uncertainty, making it difficult to determine a consistent pattern.
- Trend**:** This suggests that in older individuals, at lower quantiles, exposure may be associated with a slight increase in LDL, while at higher quantiles, exposure appears to be associated with a decrease in LDL. However, given the wide credible intervals and their overlap with zero, these trends should be interpreted with caution.
- Credible Intervals: The credible intervals remain wide across all quantiles, particularly at the extremes, and consistently cross zero, indicating significant uncertainty and preventing strong conclusions about the relationship between PFAS/metals exposure and LDL in older individuals.

Summary

The results indicate a mixed pattern in the association between PFAS/metals exposure and LDL across younger and older individuals, with no clear or consistent trend emerging. However, wide credible intervals crossing zero in both age groups suggest a high degree of uncertainty, making any observed patterns inconclusive.

- Older Age Group (First Plot): The effect estimates fluctuate across quantiles, with some positive and negative associations observed. However, the large credible intervals crossing zero at all points indicate substantial uncertainty, suggesting that the relationship between PFAS/metals exposure and LDL in older individuals is highly variable and not strongly supported by the data.
- Younger Age Group (Second Plot): The effect estimates **also** fluctuate across quantiles, with some variation around zero but no clear increasing or decreasing trend. Similar to the older group, the wide credible intervals overlapping zero suggest a high degree of uncertainty, preventing strong conclusions about the impact of PFAS/metals exposure on LDL levels in younger individuals.

Overall Interpretation:

The findings present a mixed and highly uncertain picture, with no strong evidence of a consistent relationship between PFAS/metals exposure and LDL levels in either age group. The variability in estimates across quantiles, combined with wide credible intervals, highlights the need for caution in interpreting these results. Further research with larger samples and refined exposure assessments may be necessary to clarify potential age-specific effects.

LDL Risk summary for 10th percentile age effect modifier	LDL Risk summary for 90th percentile age effect modifier
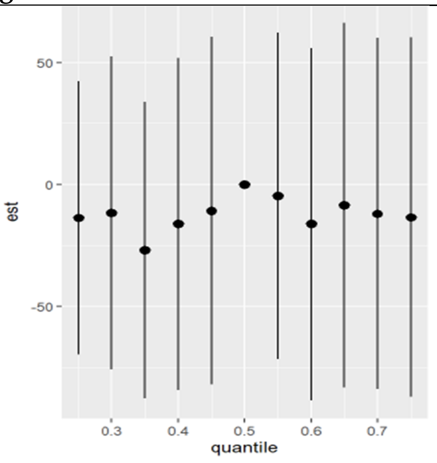	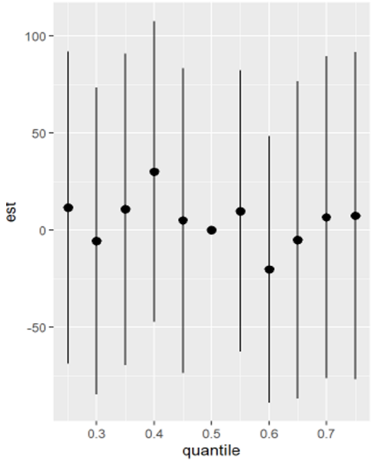

Supplementary Figure 11. The Overall exposure effect of combined PFAS and Metals on LDL examined at 0.25 – 0.75 quantiles of exposure as compared to the 0.5 quantile.

BKMR-CMA was also used to estimate the direct and indirect effects of PFAS and Metals mixtures on LDL through the DII level according to age (Supplementary Figure 12). The results were as follows:

Left Plot: 10th Percentile of Age (Younger Age Group)

- TE (Total Effect): The total effect, which combines both direct and indirect effects, is negative, but the wide credible interval crossing zero suggests considerable uncertainty.
- NDE (Natural Direct Effect): The NDE **is** positive, but the large credible interval crossing zero indicates high uncertainty, making it unclear whether there is a meaningful direct association between PFAS/metals exposure and LDL when DII is controlled for.
- NIE (Natural Indirect Effect): The NIE is negative, suggesting that DII may mediate a slight decrease in LDL due to PFAS/metals exposure. However, the wide credible interval crossing zero highlights substantial uncertainty.
- CDEs at Different Quantiles of DII (10%, 50%, 75%): The Controlled Direct Effects across these quantiles are below 20, suggesting a modest positive association between PFAS and metals exposure and LDL across exposure levels. However, all credible intervals are large

and cross zero, indicating that this trend is highly uncertain and should be interpreted cautiously.

Right Plot: 90th Percentile of Age (Older Age Group)

- TE (Total Effect): The total effect is negative, suggesting that combined exposure may be associated with a slight decrease in LDL. However, the wide credible interval crossing zero indicates significant uncertainty, meaning no clear association can be established.
- NDE (Natural Direct Effect): The NDE is approximately -40, suggesting a potential negative association between PFAS/metals exposure and LDL levels in older individuals. However, the large credible interval crossing zero highlights considerable uncertainty, making this effect inconclusive.
- NIE (Natural Indirect Effect): The NIE is approximately 35, suggesting a potential positive mediation effect, where DII may partially mediate the relationship between PFAS/metals exposure and LDL, contributing to a modest increase in LDL levels. However, the wide credible interval crossing zero indicates that this effect is highly uncertain, and further investigation is needed to clarify the strength and direction of this relationship.
- CDEs at Different Quantiles of DII (10%, 50%, 75%): The Controlled Direct Effects across these quantiles are close to zero, suggesting little or no negative association between PFAS/metals exposure and LDL across exposure levels. While this could suggest that elevated PFAS/metals exposure is linked with reduced LDL levels in older individuals, the wide credible intervals crossing zero indicate that this finding is highly uncertain and should be interpreted with caution.

Summary

PFAS and metal exposure may influence LDL levels more prominently in younger age groups**,** suggesting that age could potentially modify the impact of these exposures on LDL. However, the wide credible intervals crossing zero indicate substantial uncertainty, making this relationship inconclusive.

Additionally, dietary inflammation (DII) does not appear to play a meaningful mediating role in this relationship. The effect of PFAS and metals on LDL does not substantially change across different levels of DII**, and** adjusting for DII does not reveal any strong or consistent patterns in LDL levels related to PFAS and metals exposure.

Overall, while there is a potential trend suggesting that the influence of PFAS and metals on LDL may diminish with age, the high degree of uncertainty prevents strong conclusions.

**LDL Risk summary for 10th percentile age effect modifier**	**LDL Risk summary for 90th percentile age effect modifier**
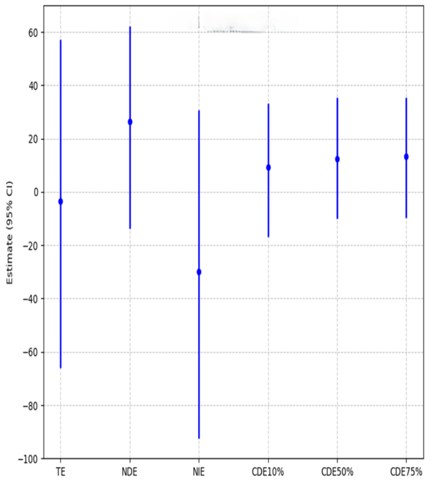	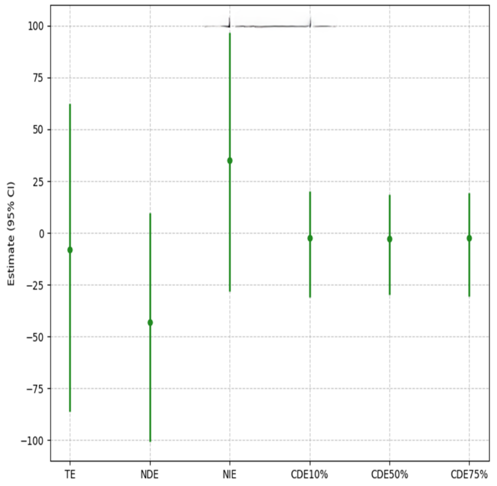

Supplementary Figure 12. Causal Mediation analysis assessing the combined effect of PFAS and Metals on LDL with DII as mediator.
